# Blame the Patient, Blame the Doctor or Blame the System? A Meta-Synthesis of Qualitative Studies of Patient Safety in Primary Care

**DOI:** 10.1371/journal.pone.0128329

**Published:** 2015-08-05

**Authors:** Gavin Daker-White, Rebecca Hays, Jennifer McSharry, Sally Giles, Sudeh Cheraghi-Sohi, Penny Rhodes, Caroline Sanders

**Affiliations:** 1 National Institute for Health Research (NIHR), Greater Manchester Patient Safety Translational Research Centre, Institute of Population Health, University of Manchester, Manchester, United Kingdom; 2 Department of Psychology, National University of Ireland (NUI) Galway, Galway, Ireland; 3 NIHR School for Primary Care Research and Manchester Academic Health Science Centre, University of Manchester, Manchester, United Kingdom; Supportive care, Early DIagnosis and Advanced disease (SEDA) research group, UNITED KINGDOM

## Abstract

**Objective:**

Studies of patient safety in health care have traditionally focused on hospital medicine. However, recent years have seen more research located in primary care settings which have different features compared to secondary care. This study set out to synthesize published qualitative research concerning patient safety in primary care in order to build a conceptual model.

**Method:**

Meta-ethnography, an interpretive synthesis method whereby third order interpretations are produced that best describe the groups of findings contained in the reports of primary studies.

**Results:**

Forty-eight studies were included as 5 discrete subsets where the findings were translated into one another: patients’ perspectives of safety, staff perspectives of safety, medication safety, systems or organisational issues and the primary/secondary care interface. The studies were focused predominantly on issues seen to either improve or compromise patient safety. These issues related to the characteristics or behaviour of patients, staff or clinical systems and interactions between staff, patients and staff, or people and systems. Electronic health records, protocols and guidelines could be seen to both degrade and improve patient safety in different circumstances. A conceptual reading of the studies pointed to patient safety as a subjective feeling or judgement grounded in moral views and with potentially hidden psychological consequences affecting care processes and relationships. The main threats to safety appeared to derive from ‘grand’ systems issues, for example involving service accessibility, resources or working hours which may not be amenable to effective intervention by individual practices or health workers, especially in the context of a public health system.

**Conclusion:**

Overall, the findings underline the human elements in patient safety primary health care. The key to patient safety lies in effective face-to-face communication between patients and health care staff or between the different staff involved in the care of an individual patient. Electronic systems can compromise safety when they override the opportunities for face-to-face communication. The circumstances under which guidelines or protocols are seen to either compromise or improve patient safety needs further investigation.

## Introduction

There is a long standing literature on the “iatrogenic” harms that can arise from medicine or health systems. In the 1970s, Ivan Illich argued that technological medical processes cause more harm than good [1]. Since then, increasing research has concerned patient safety from a variety of theoretical perspectives, although these have tended to focus on hospital medicine [2–4]. In inpatient settings, treatment and care is usually administered directly by health care workers. However, in ambulatory settings, including primary care, treatments such as prescribed medications are usually administered by patients’ themselves. For this and other reasons, patient safety has particular features in primary care including diagnostic uncertainty, the management of polypharmacy, a culture of continual organisational change and the potential for “information overload” [5].

Several previous studies have set out to present taxonomies or classifications of the types of medical errors found in family practice [6–12]. Based on a synthesis mainly of studies of self or incident reports by clinicians, Elder and colleagues distinguished between “preventable adverse events” (related to delayed or missed diagnosis and incorrect, omitted or delayed treatments or preventive services) and “process errors” (e.g. related to clinical or procedural skills and communication or administrative factors) [6]. In a study of self-reported errors by physicians during a randomized controlled trial of computer reporting systems, errors were seen to arise either from dysfunctions in health care systems or deficits in skills or knowledge [7]. The sub-categories found largely reflected those found in Elder’s synthesis [6]. In an international comparison of primary care medical errors in 6 (mainly English-speaking) countries, 7 categories of error were found which again reflected those seen in the previously cited studies (e.g. “office processes”, communication, treatment and clinical knowledge) [8].

More recently, analysis of error reports made by primary care physicians in the USA [9] and Japan [10] suggests that they most frequently involve misdiagnosis or procedural complications, although errors concerning communication, medicines [10] and wider organizational or systems issues [9] are also highlighted. A survey of Swiss primary care physicians and nurses identified similar concerns, although in contrast to the other studies, medication safety was mentioned more frequently than communication, procedural or systems issues [11]. A US community survey of the perceived harms caused by medical mistakes found emotional, financial and physical consequences for patients. However, mistakes raised by patients were broader than clinical issues and also incorporated unmet expectations, violations of trust and criticism of doctors’ manner or attitudes [12].

Whilst the studies above are useful for delineating the types of errors that might be found in primary care, they say less about the social or cultural context in which errors arise (e.g. due to the behaviour or characteristics of patients and staff or processes in the organisation of care) and what might be done to reduce threats to patient safety. Traditionally, a “measure and manage” approach has been adopted in patient safety research, but this “has a tendency to neglect or downplay important issues associated with professional practice, teamwork, culture and organizational complexity” [13]. Mixed-methods studies have been employed to examine patient safety in relation to specific technological innovations or procedures in primary care, including management of test results [14,15] use of electronic health records (EHRs) [16] and e-Prescribing [17,18]. Collectively, these studies are located at the interface of technological and social processes or practices, including workflow [15] and organizational safety culture [14]. Use of electronic systems can itself contribute to safety failures resulting from new errors associated with the technology itself, such as “alert overload” [18] or accidentally selecting the wrong drug from a pick list menu [17]. A task analysis of physicians’ use of EHRs found that they interfered with patient-doctor communication [16].

Qualitative research, in the form of semi-structured interviews, observation studies or focus groups, is commonly used as a means of exploring experiences and perceptions in Health Services Research. Such approaches have been used in hospital-based studies of patient safety; allowing for in depth exploration of the ways in which organisational or social processes interact with the potential to bring errors or harms [19–21]. The aim of this study was to use meta-ethnography [22] to synthesize the *findings* (i.e. the interpretations of the authors of primary studies) of published qualitative research concerned with patient safety in primary care, partly to develop an analytical framework for a longitudinal qualitative study concerned with the management of multimorbidity [23]. The objective was to develop a conceptual model that incorporated features that would only become apparent when the findings of individual studies were put together or compared with each other. However, in the end result, development of “third order interpretations” [24] was not always achieved through the translation and synthesis of findings. More important insights arose from those occasions where groups of findings about particular topics appeared to contradict each other, or were “refutational” [22].

## Materials and Methods

The study described in this article used meta-ethnography as a means of synthesizing the findings of studies as originally designed for use in Education research [22] and later developed for use in health services research (incorporating methods of searching and study appraisal borrowed from the science of systematic reviewing) [25]. Meta-ethnography is an interpretive method that involves iterative re-organisation of the findings of included primary studies until they can be “translated” into one another in a meaningful and coherent way [22]. Following the “translation” of study findings, key concepts or “metaphors” (i.e. the words that authors’ themselves use, rather than study participants) there follows construction of refutations, lines-of-argument and/or higher order concepts that lead to the expression of a synthesis that in some way moves beyond the findings contained in reports of individual studies [25]. Noblit and Hare suggest that there are seven stages to meta-ethnography: topic selection, selecting and finding studies, reading the studies, determining how they are related, translating the studies into one another, synthesizing the translations and expressing the synthesis [22]. Determining how studies are related could involve breaking them down into those examining similar issues, especially when a large number are found [25]. For example, in a meta-ethnography of qualitative studies of medications adherence, 38 articles were grouped into types of medicines [26]. When it comes to translating the findings of studies into one another (within groups or subsets), one approach involves arranging articles in chronological order of publication and using the findings of the first as an ‘index paper’ against which those of all the others are compared [27].

The team consisted of experienced qualitative researchers all working in the fields of primary care patient safety and/or patient experiences of chronic illness or long-term condition management. A protocol was designed in advance of the study. The study used a simple search strategy so as to allow the comparison of Google Scholar and bibliographic databases as means of locating qualitative studies for a meta-synthesis (see discussion). Team and sub-team meetings were held throughout the searching, appraisal, translation and synthesis stages of the study and differences of opinion were dealt with through discussion.

### Inclusion Criteria

We included published reports of studies of patient safety in primary care employing qualitative methods of data collection and analysis. Articles reporting mixed-methods studies would be included where substantive qualitative findings were presented, i.e. consisting of groups of themes or concepts developed from interviews or observations and illustrated by quotations taken from participants or field notes.

### Exclusion Criteria

The following studies were excluded: mixed-methods studies without substantive qualitative data or findings; studies not focused on patient safety; studies not located in primary care; studies focused on care provided either in patients’ own homes (including care homes); and, studies evaluating interventions to improve safety. At synthesis, two studies were excluded which did not fit and findings from studies concerning doctors’ attitudes towards error reporting were excluded, although other findings from these articles were retained (see below).

### Search Strategy

Google Scholar and the bibliographic databases Medline, Pubmed, CINAHL, Embase and Web of Knowledge were searched using a simple search strategy:

("patient safety" OR "adverse event") AND ("primary care" OR "general practice" OR "family practice") AND ("qualitative" OR "ethnographic" OR "ethnography" OR "semi-structured" OR "focus group")

The search strategy was piloted in Google Scholar, which appeared sensitive to the length of the search string and the number of terms incorporated. Google Scholar and the other databases were searched independently by different workers (GD-W and RH) in order to allow a blinded comparison of the relative yield of each. One worker (SG) searched two specialist online repositories of publications of patient safety research (http://www.patientsafetyinstitute.ca and http://psnet.ahrq.gov/) and three workers (RH, JM, PR) conducted electronic “hand” searches of the journals BMJ, British Journal of General Practice, Annals of Family Medicine and the reference list of an online report found at one of the online repositories [28]. The reference lists of any articles included in the synthesis were “back searched” for additional references and the “cited by” feature of Google Scholar was used to “forward search” for more recently published material. Backwards and forwards searching was conducted contemporaneously with quality and appraisal and data extraction. The database searches were undertaken during December 2013. Reference list and citation searching was conducted during early 2014. English language materials published in print or online up until the end of 2013 were included. Following the identification of relevant studies an assessment was made on the nature of the substance of the findings and initial scope of the synthesis.

### Identification & Abstract Screening

**Fig 1:**
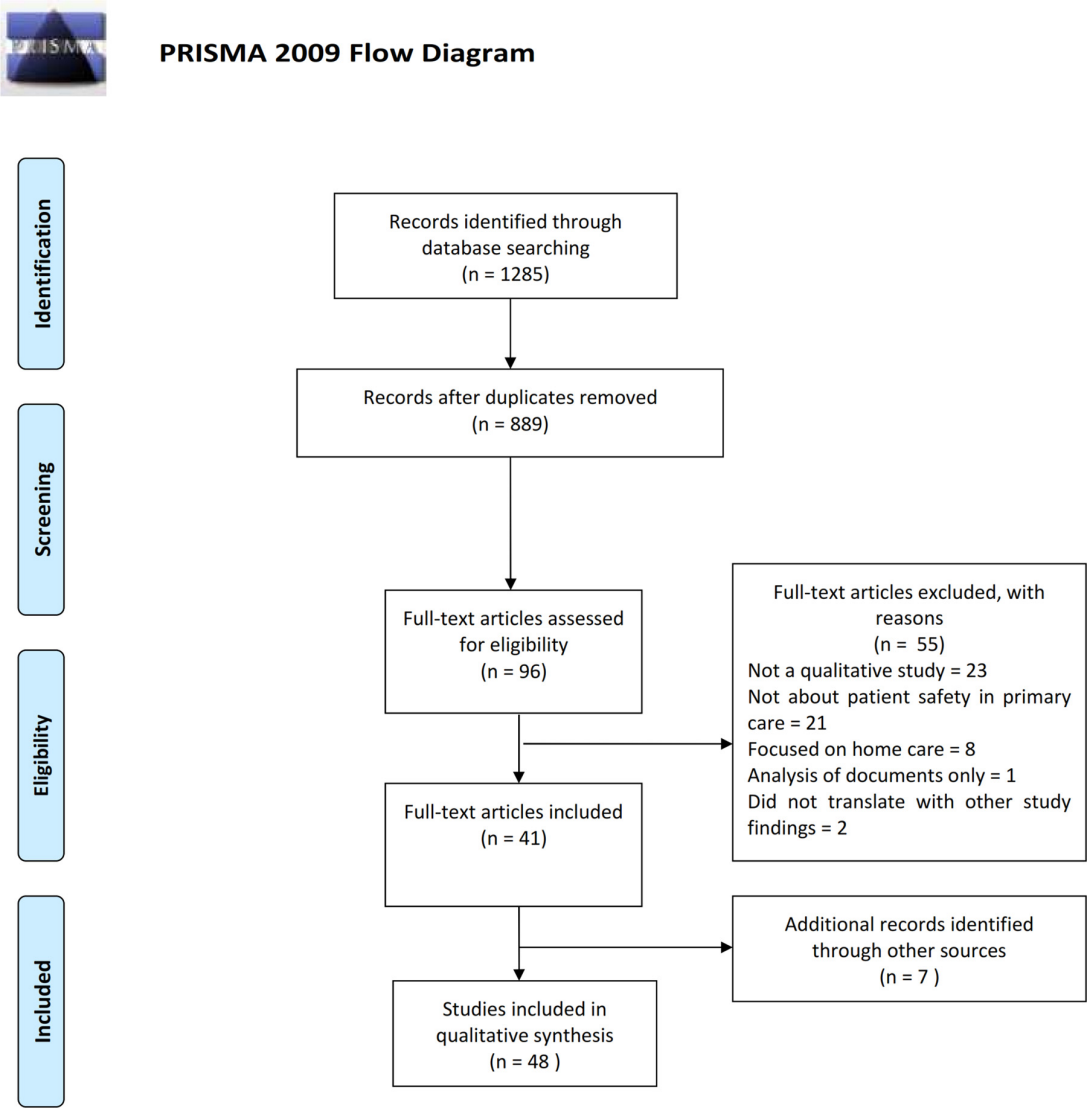
Prisma Flowchart.

Fig 1 shows the results of searching and abstract screening according to the PRISMA standards. However, in this meta-synthesis, individual workers conducted different searches blinded to each other (in order to compare the yield of different searching strategies); duplicates were removed simultaneously with initial appraisal and articles were excluded at different stages. Thus, reference to Table 1 provides a fuller picture of what was included and excluded at different stages and also shows results for searches of journals ‘by hand’ and grey literature which we conducted as primary search strategies. Although 17,200 results were returned in Google Scholar, it was only possible to screen the first 1,000 results due to restrictions imposed by the search engine (hence the significantly reduced number of search results shown in Fig 1 compared with Table 1).

**Table 1:** Searching, assessment, inclusion and exclusion of relevant articles.

	Source (Searcher)	Number	Running Total
Articles Found in initial “simple” searches	Bibliographic Databases (RH)	155	
	Google Scholar (GD-W)	17,200	
	Hand Searches (JM, PR, RH)	386	
	Web sites (SG)	37	
Following initial assessment by searcher	Bibliographic Databases	39	
	Google Scholar	70 / 1000 (see text)	
	Hand Searches	12	
	Web sites	37 (not assessed)	
Following initial appraisal and removal of duplicates	Team meeting		**76**
Following exclusion of mixed methods studies and those focused on home care	Team meeting		**62**
Articles excluded during full assessment, appraisal & data extraction	Individual reviewers / data extractors	19	**43**
Articles found in reference lists of included studies	Individual reviewers / data extractors	3	**46**
Articles found by using the “cited by” feature in Google Scholar applied to included reports	Individual reviewers / data extractors	4	**50**
Articles excluded during the translation of study findings stage	Pairs working on each subset of articles (see text)	2	**48**
**Total number of articles included in the meta-synthesis**		**48**	

In contrast to a systematic effectiveness review, where it is advantageous to locate every randomised controlled trial of interest, there is an ongoing debate about whether including large (vs. smaller) numbers of studies in a qualitative meta-synthesis affects the results [25]. Meta-ethnographies usually involve a small number of studies, although two recently published examples have incorporated 77 [29] and 52 [27] articles respectively. Some of the exclusion criteria we applied, such as removal of ‘mostly quantitative’ mixed methods studies and those focused on home care (see above), were in order to reduce the number of studies identified which were considered to be unmanageable for a meta-ethnography. Again, the iterative nature of determining what should and should not be included in a meta-ethnography is not well represented by a PRISMA flow chart, which assumes an exclusively *a priori* approach. In a meta-ethnography, the focus of the synthesis is partly determined by what is found in searches and whether it ‘fits together’ when attempts are made to translate the findings into one another.

Table 1 shows that 14 articles were excluded following initial assessment, 19 were excluded at full appraisal and data extraction and 2 were excluded during the translation of study findings and synthesis stages: 1 because it was fundamentally about doctor-patient communication with non-English speaking patients, rather than patient safety *per se* [30] and one which was focused on community development [31]. Whilst these latter two papers were about patient safety in primary care, the findings did not translate with the rest of the studies, i.e. they did not ‘fit’ in the tables of concepts (see below). By “back” and “forwards” searching from included articles, a further 6 reports of studies were located (Table 1). During the synthesis process, some aspects of three further studies concerned primarily with staff attitudes to incident reporting were excluded as they did not fit conceptually with the findings of the bulk of the studies (see below). However, these articles were not counted as “excluded” as some other findings from them (not focused solely on incident reporting) were nevertheless retained. Throughout the study, data on inclusions and exclusions was managed using Microsoft Excel. The references were managed in EndNote and data extraction forms were completed in Microsoft Word. Eventually, 48 articles were included in the final synthesis.

### Study appraisal and data extraction

Quality appraisal and data extraction were undertaken using a standardised form based on one used in a previous meta-synthesis [32] (S1 Appendix). Quality appraisal was conducted using a simple checklist of prompts, developed by others, concerning the specificity of study aims and objectives, the appropriateness of the design, the method and account of methods of data analysis, and whether the data supported the findings [33]. Given the large number of papers found, they were divided into subsets constituting studies that appeared to be broadly about the same things: patient perceptions of patient safety, staff perceptions of patient safety, medication safety in primary care, the primary/secondary care interface and “others” which were subsequently found to focus on organisational and systems issues. The work of study appraisal and data extraction was divided up amongst team members, with different researchers working on different subsets. The primary worker/s on each subset of articles read the papers in full, conducted ‘forwards’ and ‘backwards’ searches for other potentially relevant material, took the decision on what to include and exclude, and conducted quality appraisal and data extraction. The first author extracted some findings from studies involved in each of the subsets (except patient perspectives), in addition to the subset he was primarily assigned (medications), in order to maintain an overview. In each case, the whole article (including the abstract) was read and findings were extracted verbatim onto an electronic version of the data extraction form (see S1 Appendix) with page references. In most cases, participant quotations were not extracted, unless authors’ discussions of the same seemingly failed to capture the themes or concepts illustrated in the quote—in which case the quote was extracted. Thus, the focus was on authors’ interpretations of the data collected (e.g. as in “metaphors” [22]) rather than the “first order” participants’ words as presented in illustrative quotations.

### Translation and synthesis

Translation of findings from the different subsets of studies was undertaken by the same worker/s who had appraised them and extracted data into the standardised forms: patient perceptions (PR), professional perspectives (JM), medications (GD-W), organisational and systems issues—was “other”—(RH) and the primary / secondary care interface (SG & SC-S). A further worker who had not undertaken searching, appraisal and screening was used to provide a critical overview (CS). The main method involved iteratively re-organising tables of concepts or factors that best allowed the studies to “translate into one another” [22,25]. The first author was also involved in this process for every subset. Each table was re-organised three or more times (alongside group discussion) until a satisfactory means was found of expressing the findings in a manner in which they best ‘spoke to each other’. The synthesis involved close reading of each cell, row and column of the final versions of the tables in order to express concepts, themes or “metaphors” [22] that best characterised the contents. Sometimes this process resulted in conceptual development that went beyond the original findings and sometimes it did not [25]. These tables of combined second order concepts and “third order interpretations” [24] were then compared and used to develop a conceptual model that best described the main factors and issues relevant to patient safety in primary care. The synthesis was principally expressed as a descriptive narrative of the nature and findings of the included studies, with a line-of-argument pertaining to the (limited number of) third order concepts that were developed from translating the findings into one another.

## Results: Study characteristics, quality of methodological reporting and translation of findings

### Subset 1: Articles focused on patients’ perceptions of patient safety in primary care

#### Overview

Six included studies were concerned solely with patient experiences or perceptions of patient safety in primary care, although one study in this group also included hazard reports and focus group data collected from patients’ physicians [34]. The reviewer was concerned that that the latter study only included 14 patients. All of the others relied on semi-structured interviews with patients (whether recruited in community or service settings). The earliest paper was used as an index paper for the purposes of translating the study findings. It described a study of patients’ perceptions of the most important errors or harms in primary care [35]. Whilst the worker who appraised the study described the quality and reporting of study methods as ‘excellent,’ it was also noted that the nature of the sample (29/38 of whom were African Americans) and the way the data were collected and analysed may have led to issues of discrimination being magnified. However, the study was the only one in the subset to report a form of respondent validation, through “reactor panel” focus groups [35]. The findings were organised around errors related to access, communication, relationship breakdowns, technical errors and “inefficiency.” This key article for the synthesis was most useful for demonstrating how “trivial insults could eventually lead to more serious problems” [35]. A further two articles that were initially examined in the subset of papers concerned with organisation and systems (where they were found not to fit) were eventually added to this group as the findings were similar. Both consisted of reports of the same group interview study and most of the participants (64/83) were patients [36,37]. Thus, this subset consisted of a total of 8 studies.

Research methods and reporting thereof were considered to be ‘excellent’ in one further article other than the index study [38] and acceptable in 6. One study was considered ‘unacceptable,’ with substantial weaknesses in reported methods of data collection and analysis [39]. Most (5/8) studies were undertaken in the USA, one was conducted in the UK [40] and two articles constituted reports of a single study conducted in New Zealand [36,37]. In the case of one study, the reviewer was concerned about the apparent distinction between the classification scheme used by the researchers (access, safety, relational and management continuity) and the reported patient accounts [40]. Patients were seemingly not directly asked about safety, so the analysis seemingly relied on the researchers’ interpretations of the data in this respect, rather than the patients.

With the exception of the index paper, despite a broad concern with patient experiences or perceptions of safety issues, each had a particular focus: the effects of errors on subsequent interactions with healthcare [38]; the perspectives of elderly patients [34]; patients’ preferences in respect of communication of “normal laboratory test results” [41]; the views of residents of urban areas [39]; and the perceptions of error in the management of long-term conditions [40]. The test results study was seen to have an especially narrow focus and included some descriptive statistics [41]. The two articles that were originally assessed within the organisation and systems subset, but found not to fit (see above and below), reported the results of a study that was concerned with a taxonomy of the relative threats to safety in primary care and means of reducing them [36,37].

#### Findings

The different focus of the studies is reflected in Table 2. Thus whilst findings from 5 were concerned with characteristics, issues or processes seen to adversely affect patient safety in primary care, the Elder *et al*., study [38] didn’t seem to fit with the others but rather formed the kernel of a different group of findings which were about processes or actions that were seen to maximize the impact of harm once an error had occurred. Only two other studies contained findings in this respect: the key index paper [35] and the UK-based study, which seemed to really be about continuity of care rather than patient safety [40], although continuity of care is recognised as a safety issue for patients [42]. Only three of the studies, including Elder *et al*. [38] considered the ways in which patients might protect themselves against safety incidents. Most of the pertinent findings of the New Zealand-based study derived from the second report of the findings (and not the taxonomy) and contributed heavily to the row of Table 3 concerned with “actions to promote safety” [37].

**Table 2:** Translation table—Findings from qualitative studies focused on patient perceptions of safety in primary care.

	Patients	Staff	Doctor-Patient Communication	System
Adversely Affects Safety	“Physical limitations common to the elderly”; “Difficulty keeping track of medications”; “memory limitations” [34]	“Relying on the patient for information transfer” [and] “the patient’s memory” [34]	“Many healthcare encounters” [34]	“lack of follow-through and confusion within the office”; No awareness “lacked a standard procedure for communicating test results” [41]
	“Patients' preferences” [41]	“physicians that are inadequately trained or who have not maintained their competence” [35]	“Lack of communication” “responsiveness and interactive feedback”; “a message left on an answering machine was not appropriate”; “it can take days for successful telephone contact between providers and patients” [41]; “lack of feedback”; “The patient is just left guessing”; “Lack of communication and integration between systems”; “insufficient communication” [34]; ‘I was about to die from nervousness and waiting’ [39]	“access breakdowns created by long waits for appointments”; “Trying to get through”; “Checking in”; “Waiting to be seen” [35]; “Time delays and waiting; “Availability and accessibility of care” [34]; “the ‘gatekeeping’ role of health service staff” [40]; “frustration with waiting”; “substantial unmet needs for appointment access”; “difficulty accessing providers for urgent problems; “Difficulty contacting physicians between appointments” [39]
	“do not discuss with their provider” [41]	“relationship breakdowns involving rude staff, disregard for patient concerns, and racial bias”; “prejudice”; “Insensitivity and miscommunication” [35]; “The doctor’s apparent failure to take the patient’s concerns seriously”; “the doctor appeared to be uninterested in the patient’s problems” [40]; “inattention” [39]	“notification by receptionists, who they felt were not knowledgeable enough to answer questions”; “Patient's privacy and assured confidentiality”; “possibility of a breach in this trust.” [41]	“offices being too busy”; “the unintended consequences of managed care” [35]; “under-staffing and underfunding” [39]
	“Attendance errors”; “adherence errors”; “patient memory errors;” “mindfulness errors”; “Misjudgements include such errors of assessment as a failure to check, monitor, or record … a wrong judgment … and unrealistic expectations by patients who expect too much or too little of themselves or others”; “knowledge errors, such as low literacy; comprehension errors; and errors of logic” [36]	“Insufficient medication information provided.” [34]	“the amount, content, and method of patient communication. … saying too little or too much … What they talk about may be inaccurate or unhelpful … communicate unclearly, with disrespect, or artfully. Forms of artfulness include dishonesty, pretence of sickness, and manipulating the system” [36]	“insurance coverage and ability to pay” [39]; “Cost concerns” [34]“Information not transferred or lost”; “Incomplete or scattered information. Subjects described instances of missing, incomplete, or scattered patient records”; “patient information in the EHR was scattered, incomplete, or inaccurate. However, subjects were very confident about the completeness and accuracy of those same electronic records” (“**hidden hazards**”)
				[34]“Many healthcare professionals” [34]
Unsafe Practice	“The most common emotional response to experiencing preventable problems was anger”; “Mistrust”; “A sense of resignation”; “a partial or total avoidance of the system”; “navigate around the parts of the system where the problems occurred, such as avoiding the telephone or the office staff;” “avoided their current doctor, office, or hospital by switching to another one” [38]	“stories of disrespect or insensitivity” [35]; “perceived lack of empathy on the part of doctor”; “an apparent failure to respond to the patient’s concerns leads to a negative emotional reaction” [40]; “experiencing a preventable problem affects trust, resulting in an association of mistrust with health behaviors” [38]	“a breakdown in interpersonal communication”; “the lack of effective interpersonal care may have compounded a possible prescription error and exacerbated both physical and emotional “harm” to the patient” [40]	“Problems of access may also impact on relational continuity if unfamiliar sources of care must be utilised” [40]
			“seemingly trivial insults could eventually lead to more serious problems and that even near misses could cause anxiety and diminished trust.” [35]	
Actions To Promote Safety	“written notes and printed information sheets to help them remember” [34]; “own use of skills in managing healthcare encounters”; “astute observers of health-care systems and sophisticated participants in healthcare interactions”; “understanding of the complex factors that create frustrating conditions” [39]	“Patients appreciated a ‘positive attitude’ and valued good listening skills”; recognition, personal assistance and respect. Several patients were pleased when greeted by name. … Subjects regarded mutual respect between staff and patients as part of good care”; “Patients recognized physicians do not have unlimited time for visits and appreciated any indication that a physician had expended extra time or effort for them.” [39]	“if we respect the doctor, then they will respect us”; “bringing family members to a visit improved care”; “Participants identified use of specific skills in these areas such as partnering, encouragement and recognition of patients’ emotional states” [39]; “reliance on others” [34]; “timely recognition of mishandled or misplaced results will increase the practice's ability to correct or mitigate an error” [41]	“always being notified of results, timeliness, details of the results, responsive and interactive feedback, who should provide the notification, convenience, and assured security / confidentiality” [41]
	“Anticipation,” “Attend to details,” “Accommodation … to adjust to the system”; “Acquire knowledge,” “Actively comm-unicate,” “Attend to emotions,” “Advocacy behaviors,” “Spoke up for themselves” [38]	“by offering patients (and their informal caregivers) oral and written information about the importance of what they are being asked to remember and by using relevant routines such as memory aids”; “improve their own personal care delivery, especially communication skills so that patients feel safe to speak openly, ask them questions and return for future care”; “[GPs] need to ‘come out of their offices and into their communities’. … such engagement could, … address a tendency for general practice to detach patients from their natural environment” [37]	“Relationship-building, for example through improvements to communication, can enhance the ability of patients to … trust a professional enough to admit to non-adherence” [37]	
	“Relationship-building, for example through improvements to communication, can enhance the ability of patients to ‘ask family for help’ (teenagers group); “Patients were encouraged: to know their ‘neighbours’, … to access the strengths of social networks such as family and friends; and to increase their level of education so that they can help themselves and others” [37]			

**Table 3:** Opportunities and threats for patient safety in primary care (staff perspectives).

	Threats to Safety	Reducing Error
Primary care staff characteristics	Distracted by “personal concerns” [48]; “inability to balance work and family life affects morale and concentration”; “Women physicians talked more about time pressure”; “Some minority physicians reported difficulty” [44]	“resist outside distractions” [48]; “Beyond the job description” [44]
	“changing role of GPs”; “performance variation between practices and GPs”; “lack of computer skills” [52]; “inadequate support as house officers”; “lack of medical knowledge’” [48]	“good medical knowledge as an important factor” [53]
Primary care staff behaviour	“ignoring gut feelings”; “competence confidence arrogance or risk taking or not risk caution”[50]; “Expectation of mediocrity”; “Personal resistance to change”; “People do not follow protocols”; “They all want to do their own thing … system, their own way of charting, their own way of documenting, everything” [46]; “unwillingness to adopt new practice methods and undertake training” [52]; “cling to clumsy or untenable practice ‘…it works for me’ “[47]	“resist pressures from other physicians to change an initial impression” [48]
	“I got so excited about all the horrific injuries that I didn’t tend to business”; “inexperienced …and panicked’” [48]; “If I had been on top of it and had made the right choice in the first place, she probably wouldn't have died” [49]	“‘safety netting was pretty major’ and as a consequence, GPs went to great lengths to set one up if necessary” [51]; “medication review”; “junior GPs querying potential diagnostic errors”; “extensive computer training”; “staff training”; “sharing of knowledge and learning” [52]; “strategies for updating knowledge” [57]
	“Communication problems, poor quality note-taking, and deference to patient notes written by colleagues” [52]	“be more aware that anger can cloud a judgement” [48]
	Errors due to “anger, usually directed at the patient or the patient’s family“; “Error due to their attitude towards the patient, either dislike (6 cases) or unusual fondness (7 cases)” [48]	“resilience factors of preparedness and awareness” [47]; “refer sooner if faced with a similar problem”; “be more aggressive diagnostically with similar patient”; “broaden the differential diagnosis” [48]; “learning about common presentations with a focus on critical cues”; “learning to deal with uncertainty” [51]
“deal with immediate problems only, rather than the usual holistic approach’; “instantaneous formulations”; “search for one or two critical cues”; “focus on red flags”; “‘dividing people into two groups…: is it serious or potentially serious or not?’” [51]; “tendency to be misled by a normal finding, which overshadowed other signals that the patient had a significant illness” [48]; “focus on the natural history of disease and expected response to treatment” [50]	
Patient characteristics	“‘some patients who frequently have symptoms’”; “Lack of social support“; “overstrained/over-protective caregivers”; “the cultural background of the patient as a factor contributing to hospitalization”; “insufficient language skills by patients” [56]Older patients “difficult because of the complex nature of multiple pre-existing diseases” [51]; “needs are more. …These are sick people” [44]; “increasing complexity of cases” [52]; “psych-iatric comorbidities and substance abuse” [53]	
Patient Behaviour	“patient-related medical errors”; “Nonadherence to prescribed medications by patients… ‘He ignores medical advice!’”; “patients or their care-givers had delayed seeking help, which had resulted in the hospitalization” [56]	“promoting the own responsibility of the patients is important” [53]; “responsibility for monitoring resides with the patients” [45]
Doctor-Patient Communication	“communication problems” [46]; “Poor communication” [44]; “ignoring or misinterpreting the predictive value of critical information coming from the patient” [50]; “‘If we would have asked her more frequently: How are you doing?—the classic opening question—we may have found that she is in serious trouble and needed far more help than she declared’” [56]	“need to be circumspect when responding to patient needs” [50]; “‘I’d relieved the patient’s anxiety that was the main issue at stake’”; “training needs on how to deal with patients, stressing the need to develop rapport despite the intense time pressures” [51]
	“difficult to work out whose agenda they had to deal with, the patient's or concerned relatives or carers” [51]; “Patient expectations” [46]; “patient wishes or anticipated patient wishes”; [48]; “patient’ fearfulness as a crucial feature of some hospitalisations” [56]; “previous consultations which had set her up as a particular kind of person” [50]; “In most cases, GPs formed instantaneous diagnoses…the patient's appearance” [50]; “‘I guess his age, relatively young … I didn’t think of [cancer]” [48]	“Medical records played an important part in the communication between different employees in the practice” [53]
Organisation of care	“do I want to send a frail, elderly lady up to the hospital on a Friday afternoon when it would be mayhem” [50]; “to convince these guys to drive an hour and a half to go to [city] to get the CT scan … and then try to convince the physician that I thought this person needed a CT scan… you know, they’d say, “Yeah, right, doc” [48]	" ‘In a perfect healthcare setting would be timely access to a physician, appropriate evaluation, proper medication and compliance by the patient and also appropriate laboratory investigation and follow-up ‘ ";" ‘getting the most up-to-date evidence-based care’ "; “Proper follow-up and tracking of patients" [54]
	“laboratory tests performed in other settings that do not send results back” [45]; “problems exchanging accurate, complete and timely information with external settings” [53] “Poor communication between primary care clinicians and sub-specialists or diagnostic facilities” [44]	“electronic availability of results from investigations undertaken in hospital”; “complaints to the provider were necessary” for reductions in time for results to be returned; “communicating with secondary care to check data quality” [52]
	“felt hurried”; “quitting time phenomenon” [48]; “‘Let’s give some care to as many people as we can versus let’s give good care “[44]; “there's so many balls in the air there's a chance for one of them to drop’ “[45]; “distractions and volume of work” [54]; “fire fighting” [51]; “time constraints”; “competing work place priorities” [57]	“‘systems have to be simple or people don’t do it’” [46]
	“stressed costs and funding” [46]; “resource availability”[44]; “laboratory monitoring requires an inordinate amount of unreimbursed time” [45]; “skills or services required … are not always available or reimbursed “[56]; “duty to the patient despite financial pressures”; “uncertainties about future funding” [52]	“steps in the testing process”; “vast majority of testing process safety procedures were created by individuals”; “support and involvement of the entire organisation” [47]; “Collegiality”; “Teamwork” [44]; "The idea of teamwork is hugely important” [54]; “multidisciplinary partnerships with other staff to ensure safety” [52]
	“variation both between practices and within practices” [47]; “frequent policy changes” [52]	“‘a pharmacist that it is a safety net for them’” [44]improved staffing” [46];“better documentation”; “regular review of existing measures”; “local selection of measures” [52]
	“few examples of systematic office-wide organizational processes for testing process safety”; “none of the offices had written protocols for all of their testing procedures”; “physicians and staff at every office …described tasks and processes that were totally incorrect” [47]; “Policies and procedures, or the lack thereof, also affect quality” [44]; “No widely disseminated or agreed-upon guidelines regarding laboratory monitoring” [45]; “procedures, processes, workflow and standardization" [54]; “Not following procedures, inadequate systems, communication problems, and lack of standardization were most commonly mentioned” [46]; “insufficient communication between PCT and practices” [52]	
	“Process barriers included costs, sources of ideas, external supports, the work environment, feedback, and staffing changes” [46]	
	“physicians and staff tended to work around systems problems rather than try to solve them”; “top-level commitments” [47]; “management”; A lack of a safety culture [52]; “No perceived consequences” [46] (cf. “fear of punishment” [52])	
	“Medication lists are one aspect of electronic medical records that are particularly error-prone” [44]; “the frequent warnings of the computerized medication system, which often were not read carefully” [53]; “EHR alert systems … found to be annoying and cumbersome”; “alerts arose too frequently and desensitized the doctors into ignoring them”; “complicated navigation of multiple screens on a computer system”; high volume of computer … alerts during consultations”; “hardware breakdowns”; “technology changes too quickly”; “poor data quality and accuracy” [52]	
	“duplication of work”; “too many measures”; “too many clinical systems” [52]	
Professional roles	“inadequacies in medical education” [52]; GPs and PED consultants felt “pre-service education in vaccine safety and adverse event reporting was inadequate” [57]	“All GPs said they always read the incoming lab results. All practice nurses said they always checked deviating lab results with the GP” [53]
	“Administrators were more aware of the general process flow than were the MAs physicians or clerical staff” [47]	“Information systems can be very helpful if properly maint-ained and if clinic personnel are adequately trained” [44]; “it may ultimately lower your liability by having the computer system help check these rather than always relying on yourself with 3000 patients”[45]
	“Most GPs did not supervise their practice nurse concerning the advices given by telephone”; Agreement for nurse advice to be noted in medical record not always performed [53]	good learning process” [44]; “knowledge” [46]; “successful safety practice grew out of experiences with error”;“use of a learning culture” [47]; “specific training could be important for patient safety in the practice, for example in hygiene, triage or communic-ation” [53]; "In-service train- = ing would definitely help" [54]
	“time burden of laboratory monitoring was thought to be exacerbated when the responsib-ility for monitoring was ambiguous” [45]	“communication” [46]; “sharing of information and partnership among GPs in the locality, and between GPs and practice pharmacists” [52]
	“misled by the advice or the anticipated advice of other physicians” [48]	“important that everyone feels free to contribute their ideas because everyone has a different role and, maybe, just a different way they go about things.” [54]; “enough staff to adequately be available per clinician to adequately deal with the problems” [46]; “Respect”; “Aligned values”; “Leadership”; “Diversity”; “promotes trust and honesty”; “non-punitive” [44]
	“conflict with providers” [52]; “Lack of job control and inability to participate in decision making… ‘We don’t have a voice in what we are doing.’” [44]	
	“Compulsory reporting will [not] have the desired impact of reducing incidents of harm” [52]; “dissatisfaction with reporting methods” (GPs and consultants only, not nurses) [57]	
	“dependence on the commitment of practice staff to adopt new policies and data management”; “the autonomous nature of work in general practice” [52]; “staff issues” [52]	

Whether findings were about patients, staff, interaction or systems issues, they were fundamentally concerned with issues in doctor-patient communication or issues in the transfer of records between different care providers, which was the explicit focus of a different group of studies considered below. Attempts to translate groups of findings across studies did not seem to lead to the development of ‘new’ or ‘third order’ concepts, which could reflect the particular focus of many of the studies and the fact that many were fundamentally concerned with generating ranks or lists of concerns that constitute safety issues for patients in primary care. In this regard, the findings seemed more amenable to a thematic synthesis, which would probably have led—in circular fashion—back to the underlying theoretical frameworks that were focused on relationships, communication, organisation of health care and continuity of care. Thus, when comparing the findings of the studies against one another, the most useful insights seemed to come from instances where the interpretations made by the authors of individual studies were exceptional when compared with the bulk of the major concerns identified.

One important insight derived from the notification of test results preferences study, where it was found that “privacy” and “assured confidentiality” was a primary patient concern which led to suspicion of new technologies where there was deemed to be a “possibility of a breach in this trust” [41]. Whilst extracting the findings from this study, the reviewer came up with the metaphor “opacity of systems” which described how patients were ignorant of clinic policies and processes in relation to communication of laboratory results. Another finding that stood out was that a kind of blind faith in electronic health records (EHRs) appeared to contradict the fact that “patient information in the EHR was scattered, incomplete, or inaccurate” [34]. However, this latter finding would not have been realised were it not for the fact that the views of physicians’ on the patients in the included studies were also gleaned. This finding led the authors of the study to the metaphor “hidden hazards” which neatly underlines the issues related to the use of new technologies as a factor in patient safety in primary care.

Several meta-themes derived from a close reading of the articles concerned with patients’ perceptions of patient safety. The first was that greater contact with, or exposure to, health care services led to a greater knowledge of what risks there are, when and where they are likely to occur and the steps that can be taken to avoid or reduce them. It was also noted that some of the features of ‘safety’ mentioned by patients—such as being taken seriously and treated with dignity or respect—appeared to be outside of the ambit of the usual factors presented in patient safety research, which tend to focus on the competence of individuals and the integrity of technical systems, operating procedures and protocols. A further meta-concept seemed to relate to “patient safety” as a co-production of the clinical relationship. Thus, in a similar manner to the importance of an organisation safety culture [43], an important component of the clinical relationship between doctor and patient was creating a feeling of safety through respectful relationship building. The studies that were concerned with the consequences of, and responses to experienced harms [35,38], were particularly useful in highlighting the influence of past experiences on present and future health seeking behaviour, and service utilisation, and in showing how insults apparently perceived as trivial could spiral into serious harms in the midst of relationship breakdowns. Some of these insults, e.g. centring on the attitudes of health care staff, or what might be termed their ‘posture’ during clinical interactions, would not be conventionally be classified as “harms” although the findings underlined their capacity to cause the same.

### Subset 2: Studies about professional perspectives

#### Overview

Fourteen included articles were primarily concerned with staff perspectives on patient safety in primary care, making this the largest subset. The location of the studies was geographically diverse, with 6 having been undertaken in the USA [44–49], 3 in the UK [50–52] and 1 each in The Netherlands [53], Canada [54], Denmark [55], Germany [56] and Australia [57]. As with other subsets, although some papers seemed concerned with issues of error or patient safety in general terms [44,48,53,54], most had a narrower focus; the largest group concerned with issues in testing or laboratory monitoring [45–47]. The remainder were focused on specific issues: diagnostic error [50], clinical decision-making [51], incident reporting [55], patient safety measures [52], avoidable hospitalization [56], adverse events following immunisation [57] and the emotional impact of errors on physicians [49]. Study samples consisted primarily of family physicians, although two studies also included primary care nurses and office staff [53,54] and the immunisation study included secondary care staff in paediatrics [57].

Most of the studies were based on semi-structured interviews, although three used focus groups [44,46,54] and 2 used a combination of interviews and focus groups [45,48]. In contrast to the other article groups, no study was deemed to be ‘excellent’ by the reviewer and 5 were found to have serious deficiencies in quality of reporting of research methods: two had very limited information on study design and methods [44,52], another combined such deficiencies with very limited participant quotes in support study findings [53], one did not identify how the analysis had been undertaken [50] and one failed all five indicators of reporting quality [47]. One article, which constituted a short report, only included 5 respondents following an opportunistic sampling strategy and the reviewer noted that the themes presented “were similar to the questions” posed and were not supported by direct quotations from study participants [52]. However, the findings were nevertheless found useful for the purposes of the synthesis and none was excluded on these grounds. In general terms, several studies presented count, frequency or percentage data alongside thematic or qualitative findings. Whilst the studies were considered primarily qualitative in nature, quantitative data from mixed methods studies (see above) were not extracted. These features also underlined the ‘borderline’ status of many of the studies in this subset according to our quality and inclusion criteria.

#### Findings

Attempts to translate the findings of these studies into one another led to two discrete groups of findings. The bulk were concerned with either causes of error (or threats) to safety and ways or means of reducing error or safety threats. The main factors concerned the characteristics or behaviour of staff and patients; doctor-patient communication, professional roles and responsibilities, and systems issues related to the organisation of care (Table 3). The other group of findings was focused on the consequences for primary care staff when an error occured. These findings grouped into emotional consequences and incident reporting. Following the translation process, the latter findings were considered marginal to the synthesis, as they were mainly concerned with staff attitudes towards incident reporting, and are not considered further. The exclusion of these findings means that the bulk of those contained in three studies (which were largely focused on attitudes to incident reporting) are no longer fully represented in Table 4 [49,55,57].

**Table 4:** Translation of findings of studies focused on medication safety in primary care.

	Social	Technical
Characteristics of patients	“difficult and demanding” [59]; “nattering in your ear” [59]; “poor levels” of comprehension [65]; “diffic-ulty hearing” [58]; “do not remember” [58,63]; “memory deficits and multiple comorbidities” [60]; present with “routine issues” [59]; “battery of symptomology” [59]; “not sure what medication they needed” [65]	“Obstacles for adherence” [62]; “desires to continue to take” medication despite “may have long-term side effects” [60]; in older people “the benefit of preventative medicine may not exceed risks” [62]; reception-ists “more likely to make an error” as a result of deficiencies in older patients’ medication requests [65]
Relationships between primary health care staff	“reluctant to question GPs”; assumed “medication counselling from the GP”; “negative experiences”; “asymmetrical relationships” [58]; “role of their peers in maintaining safe practice”; “the extent to which both parties are willing to collaborate over safety-related issues” [66]; fear of blame [66]; “the level of professional trust that they have in each other” [66]; “benefits of developing a culture in which incidents were openly discussed and lessons shared and acted upon” [66]; “Knowledge about colleagues’ reasons for prescriptions was … difficult to obtain” [62]; “safety … assured … by an environment of effective, two way, and blame free communication” [64]; “poor communication and nurses’ ‘quasiautonomous role’”; “the importance of … being able to share anxieties or worries” [59]; ambiguous wording of hospital letters” [59]	“time taken to contact GPs”[58]; “community pharmacists lacked access to patients’ medical records”[58]; perceived “deficiencies in the performance of clinicians” [64]; GPs “had little information about medical indications for or changes to the drug list” [63]; “the real time activity and collaboration that actually unfolds around repeat prescribing, which is typically messy and unpredictable” [64]; “the difficulties of coordination between multiple institutions can lead to dire consequences” [60]; “dialogue was more direct when pharmacists were located in the same clinic” [60]; “e-prescribing led to … less conversation between the pharmacy and the prescriber’s office” [67]; “with little or no information” [59]
Comm-unication between patients and staff	A strategy “to create a feeling of safety” involved “interviewing patients about what other drugs they were taking … and asking patients to return if they felt unwell after taking the medication” [63]; receptionists mediate communication between patients and doctors [65]; “Difficulties in communicating”; telephone communication “a source of error”[65]	“pressure to turn around medicines quickly for the customer” [66]; “lack of time during consultations” [62]; e-prescribing meant pharmacists “remembered less about their patients” [67]; updating computer records can “fall through the net” [59]; “patients frequently call outside of the times allocated” [65]
Knowledge	“knowledge of the patient”; “perception of risk” “influenced by whether the GP was aware of having made an error in the past” [59]; “caution when using new, unusual or unfamiliar drugs” [59]; “many guidelines were perceived as too rigid” [62]; “anxiety appears when the GP’s conviction conflicts with either that of a specialist or the guidelines” [62]; “the organisation may have mechanisms for sharing resources and knowledge” [66]; patients with “knowledge gaps about medication”; “insufficient patient counselling about medication” [58]	“difficulties in accessing complex medical and medication histories in” EHRs; EHRs “did not link patient diagnoses and blood test results to prescribed medication” [58]; lack of evidence and information in drug alerts led to “cynicism” [61]; “some medi-cation alerts may not be supported by pharmacy data” [61]; “With as few as 3 medicines, most GPs felt that they were on thin ice” [62]; “an environ-ment that mixes drugs’ generic and trade names” [60]; “since e-prescrip-tions were sent directly to pharmacies, patients “were not reminded what medications they were being prescri-bed” [67]; “therapeutic training”; “drug knowledge and experience”; “picked up on the job” [59]; “importance of hands-on training” [59]; “severity of potential adverse drug effects” [59]
Responsibility	A tension between GP’s and patient’s responsibility for patient health [59]; locums “unwilling to take” responsibility [59]; “the locum pharmacist talks of his disconnect from the day-to-day activity of the pharmacy” [58]; “risk of disciplinary action or litigation should a patient be harmed” [66]; “a tendency to attribute blame to individuals unnecessarily” [66]; “doctor controlled and non-negotiable” [64]; reception staff “informally accountable” [64]; need “to get patients more involved in their own treatment” [63]; “feel more responsibility to elderly patients who take many different medications” [63]; GPs “felt they had another prescriber’s responsibility dumped on them” [63]; “patient as safety barrier” could “erode patients’ trust in the pharmacy” [66]; “at risk of being reported by patients for malpractice” [62]	“the GP’s signature holds considerable power” [65]; “ambiguities around the lack of a generally-recognized individual accountable for addressing ADRLLs” [60]; “no adequate system”; “their own limited ad hoc approaches”; “obscure medications” “prescribed infrequently” [60]; “signing drug lists for conditions that were beyond their competence to manage” [63]; “taking responsibility for all drugs prescribed to a patient was viewed as an impossible task” [63]; a conflict “between ‘doing the right thing’ and staying within legal boundaries” [66]; “level of trust in governance processes depends on who is administering them” [66]; governance should support “development of practice rather than sanctioning individuals or sites” [66]; “insufficient knowledge”; unfamiliar “with the potential side effects”; “assumed that they knew enough” [58]
Workflow	“hidden” work bridges the model-reality gap [64]; “non-adherence” to guidelines and systems “to address workload and minimise errors” [65]; “Tiredness and anxiety” [59]	Time pressure; constraints on space [64]; need to defer monitoring adverse drug reactions “to address more press-ing issues”; workload a “prominent barrier” [60] (& [62]); electronic prompts and reminders “interrupted … workflow and were not helpful”; “reminder fatigue” [60] (& [61]); “now more focused on fixing problems with e-prescriptions” than other matters [67]; “potential for GPs to be distracted and interrupted” [59]; “increased likelihood of error when staff were rushing”; guidelines “fall down when the surgery [is] busy”; lack of space, facilities or time for monitoring medications; system for sharing workload “introduced new stages for potential errors to occur” [65]

Some of the findings, particularly those concerning patients’ characteristics and behaviour, and the organisation of care, were similar to those in the medications subset where the vast majority of studies were also limited to staff participants (see below). The bulk of the findings concerned the behaviour of primary care staff, the organisation of care and professional roles. In these respects, and as will be considered below, some of the main issues (including clinical autonomy, responsibility and emotional engagement) appeared refutational [22] in that they could be seen to work for or against patient safety, depending on the circumstances.

Threats to patient safety were said to include an unwillingness to follow protocols and a “personal resistance to change,” with GPs preferring to “do their own thing” [46]. Thus, medical knowledge and training were identified as important factors in several studies [44,46,48,52–54,57] and “the autonomous nature” of clinical work in primary care was identified as a threat to patient safety [52]. At the same time however, “ignoring gut feelings” [50] was identified as a source of diagnostic error and in one study it was suggested that GPs should “resist pressure from other physicians to change an initial impression” [48]. More findings were concerned with the organisation of care than any other issue. The main groups of findings reflected those also considered in more detail in those groups of studies focused on medications safety and the primary/secondary care interface and are considered below. For the purposes of the findings scrutinised here, the main issue seemed to be the importance of teamwork to promote safety in the context of clinical autonomy. Reflecting the findings concerned with the organisation of care against those concerned with the behaviour of physicians leads to questions about the utility of written guidelines, procedures and protocols when GPs are not seen to follow them in any case.

In addition to medical knowledge, other characteristics of GPs put forward as threats to patient safety reflected the high stresses of clinical work in primary care compounded by poor work/life balance. The fact that these issues were seen to be more predominant in female or ethnic minority physicians [44] coupled with the awareness that resisting “outside distractions” [48] and “beyond the job description” [44] was necessary in order to reduce error, seemed to reflect unspoken assumptions about medical work as the preserve of single minded professional (males) able to devote a large proportion of their day to clinical duties and updating training.

In addition to issues concerning medical knowledge, training and clinical autonomy, the other main concepts that seemed to derive from the findings concerning the behaviour of primary care staff seemed to focus on professional and emotional engagement. Whether in relation to the roles (“expectation of mediocrity” [46]) and responsibilities of clinical staff generally, or the ways in which GPs interpreted (or failed to interpret) clinical signs and symptoms (“focus on red flags” [51]), this group of findings all seemed to speak to the dangers inherent in false assumptions, or “instantaneous formulations” [51]. An awareness that anger or fondness towards patients [48] might be a cause of error pointed towards the need for a neutral emotional attitude within an effective communicative posture. Issues of responsibility were also evident in relation to patients’ behaviour. The findings concerning patients’ characteristics and behaviour appeared marginal in relation to the majority of the findings in this subset, although there was clear consistency with similar results found in the medications subset (see below).

Following from the notion of false assumptions, the findings concerned with issues of interaction or doctor-patient communication underlined the inherent difficulties in managing competing agendas and the need to “develop rapport despite the intense time pressures” [51]. This group of findings also appeared contradictory (or “refutational” [22]) to some extent and pointed to GPs having to walk a fine line between “ignoring or misinterpreting the value of critical information coming from the patient” and the “need to be circumspect when responding to patient needs” [50]. These findings seemed to underline the importance of not accepting things at face value and raised questions about under what circumstances GPs should ignore or engage with patient or carer agendas, expectations and wishes.

The findings concerning professional roles pointed to institutional deficiencies in training and the organisation of care and again underlined clinical autonomy as a threat, with GPs possibly being “misled by the advice or anticipated advice of other physicians” [48]. Means of reducing error pointed broadly towards the importance of an organisational safety “learning” [44,47] culture, with “properly maintained” [44] systems and team training.

### Subset 3: Studies focused on medication safety in primary care

#### Overview

Ten included studies were focused on medication safety in primary care. The earliest published paper in the series, concerned with causes of drug-related hospital admission [58] was used as an index paper. It was the only study to include patient perspectives and incorporated an “organisational accidents” framework for the collection and analysis of data. The populations of the other studies were limited to primary care staff [59,60], prescribers [61], GPs [62,63] receptionists [64,65] or community pharmacy staff [66,67]. Both of the community pharmacy studies stated that they were concerned with the “sociotechnical context” of safety issues [66] or e-prescribing [67]. Half of the studies were located in the UK [58,59,64–66], three in the USA [60,61,67] and two in Sweden [62,63]. One study report was considered “excellent” following appraisal by the reviewer and also as a key paper for the overall synthesis. It consisted of an ethnographic study of receptionist input to medication safety [64]. Given these features of the studies, it was perhaps unsurprising that the main findings were disproportionately concerned with organisational and workplace issues. The translation of findings into one another was relatively straightforward, perhaps reflecting the explicit frameworks employed by some of the studies. The findings were most useful for highlighting the distinction between social and technical issues and processes in clinical work in primary care and how these processes play out against each other in ways that impact upon medication safety (Table 4).

#### Findings

Findings related to the social characteristics of patients in terms of medication hazards pointed to the “difficult and demanding” [59] nature of consultations and communication with patients with “memory deficits”,” multiple comorbidities” [60] and problems with hearing [58] and comprehension [65]. In addition, patients were characterised by primary care staff as “demanding,” “nattering” [59] presenting with “only routine issues” [59] and unsure about their own medication needs [65]. This group of findings all pointed to issues around problematic presentation and/or “problematic” attitudes on the part of staff such as stereotyping or the kind of “instantaneous” judgements seen in the preceding sub-section [50].

The social characteristics of patients created “obstacles for adherence” [62] and challenges for receptionists who were “‘more likely to make an error’ as a result of deficiencies (sic.) in older patients’ medication requests” [65]. The main technical challenges of managing medicines related to a trade-off between the risks and benefits of preventative medicine [62], controlling symptoms and the potential impact of “long-term side effects” [60]. Thus problematic presentation created system challenges for medications management in primary care.

The presentation of patients created system challenges that meant that doctor-patient communication could become a source of error. On the face of it, the lack of time during consultations appears as a prime factor here, although it was only mentioned in the polypharmacy study [62]. Receptionists were important mediators between doctor and patient, although patients frequently called outside of specified time windows and telephone communication was considered an additional source of error, given the aforementioned characteristics of patients [65]. In technical terms the findings concerned problems brought by computerised systems where records were not updated in a timely manner [59] or face-to-face contact between patients and pharmacists was reduced as a consequence [67].

Only one study explicitly reported on patient knowledge in relation to medication safety [58], as an extension of the findings above concerning problematic presentation. One of the community pharmacy studies noted that patients were no longer being reminded what medication they were being prescribed now that e-prescriptions were being sent direct to pharmacy [67]. All of the other findings in relation to clinical or medical knowledge were focused on the knowledge of primary care staff. Overall, the findings pointed to the problems associated with managing patients on multiple medications and the uncertain nature of evidence. A tension was suggested between medical autonomy on one hand and guidelines and technical systems (such as EHRs) on the other.

“With as few as three medicines, most GPs felt that they were on thin ice” [62] and exercised caution when using “new or unfamiliar drugs” [59]. Guidelines and other specialist knowledge were perceived as “too rigid” and potentially anxiety inducing when it conflicted with GPs own convictions [62]. There were numerous technical problems with EHRs in this regard, including difficulties accessing complex histories and a lack of linkages between test results, diagnoses and prescriptions [58]. “Prescriber cynicism” resulted from a lack of evidence or information related to drug alerts [61]. Knowledge of drug therapeutics was commonly “picked up on the job” with “hands on training” being seen as important [59]. One of the pharmacy studies flagged the utility of sharing resources and knowledge in order to reduce safety hazards [66]. In one study, it followed by extension of the above that GPs’ “perception of risk” and “knowledge of the patient” were “influenced by whether the GP was aware of having made an error in the past” [59].

The organisational perspective adopted in several studies highlighted the “reality gaps” between systems ostensibly set-up to mitigate hazards and translation into busy clinical settings where staff may be tired and anxious [59]. In the key study, it was noted that this “reality gap” is bridged with “hidden work,” such as that performed by receptionists [64]. Workload appeared as a prominent barrier in several studies [60,62,64]. Resource constraints in the form of pressures on time and space [64,65] were evident and there was potential for GPs to be “distracted and interrupted” [59]. In one instance, a “buddy system” set up to help receptionists with their prescription-associated workload was seen to have “introduced new stages for potential error to occur” [65]. Again, findings in this concept group more commonly reported on problems with “repeat alerts” [61] and “reminder fatigue” [60] associated with automated prompts or warnings in EHRs.

An important meta-narrative in the included studies (not always referred to explicitly) concerned GPs as the ultimate authorities in drug prescribing [64,65]. In patient safety terms, this had the potential to cause problems due to the autonomous nature of clinical practice and power. Notwithstanding their clinical power in terms of prescribing, problems arose at the primary-secondary care interface, when GPs came to be responsible for drugs that had not been prescribed by them [63]. Such drugs might constitute “obscure medications,” “prescribed infrequently” [60] for conditions that were beyond their competence to manage [63]. Thus, “taking responsibility for all drugs prescribed to a patient was viewed as an impossible task” [63]. A study of adverse drug reactions of long latency thus pointed to “the lack of a generally recognized individual accountable for addressing” such reactions [60]. Thus, in autonomous manner, monitoring depended rather on “their own limited *ad hoc* approaches” [60]. In the index study of drug-related hospital admissions, it was found that GPs had proceeded with “insufficient knowledge” assuming “that they knew enough” [58].

One of the pharmacy studies pointed to governance issues and presented everyday pharmacy work as presenting a conflict “between ‘doing the right thing’ and staying within legal boundaries” [66]. However, this model of working was associated with a “risk of disciplinary action or litigation should a patient be harmed” and “a tendency to attribute blame to individuals unnecessarily” [66]. These issues were also evident in one of the GP studies [62]. Other issues concerned a tendency for locums (whether GPs [59] or pharmacists [58]) to appear unwilling to take responsibility and the responsibilities that patients should ideally take for their own health care [63]. However, in one of the pharmacy studies it was suggested that “patient as safety barrier” could “erode patients’ trust in the pharmacy” [66].

The aforementioned perception that clinical governance processes are focused on blaming individuals for medication safety failures [66] seemingly forms one of the foundations on which primary health care workers interact with each other. We have already seen that workload, time and resource constraints underpin the everyday experience of clinical practice, and the (key) receptionist study intimated that receptionists’ were concerned about unduly bothering already busy GPs [64]. The index paper similarly reported that pharmacists were “reluctant to question GPs,” due to a combination of false assumptions around medication counselling, previous “negative experiences” and “asymmetrical relationships” [58]. In relation to practice nurses, their “quasiautonomous role” was identified as a problem [59]. In one of the GP studies, it was similarly suggested that GPs are even reticent to bother each other, with the finding that knowledge about colleagues’ reasons for prescriptions was considered essential but “difficult to obtain” [62]. As will be noted elsewhere in this article, information exchange and coordination of care between “multiple institutions” [60] is problematic [59]. Thus, whilst pharmacists lack access to patient records [58], GPs can also be kept in the dark “about medical indications for or changes to the drug list” [63].

In contrast to the above “reality” of everyday clinical work, the in depth analysis offered in the key ethnographic study of receptionists noted that patient safety was rather assured “by an environment of effective, two-way, and blame free communication” [64]. Similar findings were evident in one of the pharmacy studies [66] and in the findings of the English study of prescribing errors in primary care which referred to “the importance … of being able to share anxieties or worries” [59].

### Subset 4: Systems & Organisation Issues

#### Overview

Seven of the included studies focused on organisational or systems issues in primary care. The papers were published between 2007 and 2014. They looked at general practice computer systems [68], the use of electronic health records and e-prescribing [69], patient identifiers and identification at a walk-in centre [70,71], uncertainties in providing healthcare [72], safety systems in commercial organisations providing NHS primary care [73] and the introduction of an incident reporting system [74]. The studies were based in the UK [68,70,71,73], The Netherlands [72,74] and the USA [69]. Following appraisal, all were considered ‘acceptable’ in terms of quality of reporting of research methods, except for one which was considered ‘excellent’ [68]. The majority of the studies were ethnographic in nature, where the researchers carried out observations and interviews [70–74]. There was also an interview study with range of “key stakeholders” [68], and a focus groups study with healthcare providers [69]. No patients were interviewed in the studies in this subset. A small group of findings concerned incident reporting. Following the subset of papers concerned with staff perspectives (see above), these have been removed from Table 5 for clarity. This mainly led to the exclusion of some findings from one article which was focused on the introduction of an error reporting system [74].

**Table 5:** Translation of findings of studies primarily concerned with organisational and systems issues in primary care patient safety.

	Threat to Safety	Reduces Error
Characteristics	Lack of integrated systems “lamented” by staff [70]; “Innovations.. did not always spread quickly” [73]; “high staff turnover … caused problems for continuity of care”; “being part of a large organization, where noone locally owned the practice, meant that staff felt less valued” [73]	An “executive team … set strategic direction, developed policies and procedures, … and sought accountability from the practices”; Large organisations are “in a position to impose policies on practices and staff” and operate “a highly controlling system of surveillance and performance management” [73]
	“creating the crucially needed time to listen to one patient involves the risk of not being able to attend to other patients” [72]; “dilemmas of ‘choosing between risks’ … when the service is overstretched”; “using his intuitive knowledge to make a quick decision … cutting corners was … a way of dealing with competing priorities and shortage of time and resources … GPs felt they had to … take potentially risky decisions … occasionally things would go wrong”; “Medical assistants argued that few GPs confessed to making wrong medical decisions” [74]	
Functionality	“discrete fields and check boxes … may leave gaps in the clinical notes”; “check boxes, and drop down menus … introduces the potential for selecting the wrong items (ie, a juxtaposition error”; “danger of propagating inaccurate information … it is easy to copy and paste the error”; “using workarounds to create a better clinical record of a patient encounter because they felt … [the system] promoted inferior notes” [69]; “loss or corruption of information” when “transferring … between different” systems; “alerted about unimportant issues which then diminishes the impact of more serious alerts” [32]; “Mistyping of patients’ data” [70]; “invest time in recreating the electronic record and in doing so may miss important information or make errors” [68]“many patients … needed help with filling-in the booking-in form… They were either illiterate, from overseas and not speaking English, elderly or visually impaired”; “the system relies on the patient providing correct, accurate, ‘true’ identifiers”; “technology … does not support the cognitive work of healthcare staff” [70]	“accurate and accessible information for decision support” [68]; “information could be disseminated, and procedures and systems updated, very quickly” [73]; “drug-interaction alerts that were beneficial”; “customize common medications”; “send and receive messages … more readily and reduced provider response time”; “ability to better manage the patient’s health record”[69]
	“work longer hours … or … see fewer patients per day” [69]	“involving patients can act as an additional check to prevent serious errors” [68]
	“systems already have features … many practices are not using them”; “variability of computer skills … paucity of training”; “If … practices do not reliably code information in an accurate way, computerised safety features may not work.” [68]	
Assumptions, Illusions and Uncertainties	“a failure in one practice could damage the reputation and stability of all the practices”; “patients … antipathy towards … a commercial organization” [73]	“raising safety awareness and developing safety culture” [68]; “fit between the needs of the organiz-ation … [and] a service that patients would appreciate” [73]
	“patients who do not speak English may agree with anything you say” [70]	“receptionists would rely on their own cultural knowledge and past experience” [70]; “The wider range and greater number of identity attributes used … the more elements are provided for a correct ‘guess’ and for detection of record mix-ups” [71];
	“official [patient] identifiers … are not necessarily ‘unique’”; “given the context … we expect patient names to be unique even though they are not” [71]	Uncertainty is an “intrinsic part of their [GPs] clinical work” [74]; “professionals admitting that they do not know if their diagnostic assumptions are correct”; “medical staff … accept that they cannot know everything … they also try to reserve space for reevaluating their judgements; “They learn to cope with the vast number of possibilities by assuming they are dealing with the most common ones”; “healthcare professionals have developed repertoires for ‘living with uncertainty’ that help to specify which uncertainties they try to reduce and which ones they accept or even require”; “Uncertainty and errors … can facilitate original responses to challenging situat-ions”; “the complex dimensions of clinical work seem more to be about ‘making links’ and developing ‘experience-based knowledge’ … in specific settings” [72]
	“the correction of a too-narrow norm”; “leaving no room for error … can also eliminate errors that are not intrinsically bad” [72]	
Standardisation	“appropriateness of the information held in the drug ontology” [68];	“accurate and accessible information for decision support”; Need to “improve the underlying knowledge base”; “improvements … most likely to occur if mandated through regulations” [68]; “strongly focussed on developing … standardised reporting formats and systems”; “developing explicit and detailed systems for governance of quality and safety”; “standardization of processes and practices, simplifying tasks and enhancing training” [73]
	“trying to map a dynamic, living being … into a coherent stable entity” [70]	
	“problems … are those of insufficient information about … errors and insufficient protocols for reducing their occurrence”; “protocol gave them the illusion of knowing what safety meant” [72]; “new protocol … created additional time pressures reducing the time available for other important activities” [74]	

#### Findings

As with other translations, common findings could be translated according to perceived threats to safety or means of reducing error. In this subset of studies, categories included the characteristics of computer systems and health care systems, patient-provider interaction, patient-system interaction and provider-system interaction. The characteristics of computer systems were again highlighted as a means of either reducing or increasing the capacity for error according to design, content or functions. A lack of standardisation and innovation, dissemination, trust, and a safety culture were seen as threats to safety in healthcare systems. Thus, efforts to encourage or build these factors were thought to reduce error. The effectiveness of systems could only really be judged in the context of their interaction with the people they are used by (providers) and designed to manage (patients). Here, additional issues of efficiency were raised.

The main issues boiled down to the characteristics of systems or organisational factors, their functionality (or not) and the role of procedural standardisation (Table 5). A group of findings that appeared unique to this subset of studies was labelled “assumptions, illusions and uncertainties.” Characteristics of health care systems that could threaten safety centred on issues of workload and lack of resources, which have been highlighted in other subsets. Such concerns led to providers “cutting corners” and taking “potentially risky decisions” that were seen as unavoidable in the circumstances [74]. Healthcare organisations were seen as having the ability to disseminate innovations, and monitor and “performance manage” staff to improve safety. Yet, there existed a generalised “antipathy towards” large organisations with the capacity to implement such systems. Larger organisations were also seen as being at greater risk, as a failure in one practice might damage reputation and trust in the health system more generally [73].

The design or characteristics of computer systems were intrinsically linked with their functionality. For example, drop-down menus are designed to make data entry easier but they also make errors more likely as it is also easy to select the wrong option. Providers had to develop workarounds for systems that weren’t effective or “joined-up”. Such ways of working had the potential to introduce new errors and were often sub-optimal. For example, dictating notes “created a delay with regard to the availability of the clinical notes.” GPs felt they had to “work longer hours” or “see fewer patients per day” but other staff members found computer systems improved efficacy, for example, by enabling them to “send and receive messages … more readily” which “reduced provider response time” [69].

Whilst some safety functions of computer systems were underused, due to a lack of training, others were seen as being a threat to safety in their current form. For example, some key stakeholders reported concerns that drug alerts (also discussed above) were often inappropriate or “about unimportant issues which then diminishes the impact of more serious alerts”. Other stakeholders suggested that errors in EHRs could be identified or reduced if patients were more involved in their care [68].

Procedural standardisation was another refutational issue. On the one hand, errors were seen to arise because of “insufficient protocols for reducing their occurrence”. On the other, procedures were seen as offering a false sense of security by giving providers “the illusion of knowing what safety” means [72]. As in other subsets, workload was a concern and new protocols, developed to “minimise the possibility that such an error would happen again”, created additional time pressures for providers who already didn’t have enough time to do everything [74]. There were also concerns that procedural standardisation could “eliminate errors that are not intrinsically bad” and, in doing so limit innovation that could result in better care and “the correction of a too-narrow norm” [72]. The standardisation of both healthcare systems and computer systems results in a ‘one size fits all approach’ that also doesn’t take account of the fact that people are dynamic and often complex beings.

The findings labelled as assumptions, illusions and uncertainties, were hinted at in other sub-sets but made more explicit in the papers concerning organisational and systems issues. As observed above, providers or patients may have ‘blind faith’ in EHRs, yet it is easy to make errors when entering data. Furthermore, patients may not provide accurate or comprehensive information. The papers included in this subset that concerned patient identification [70,71] highlighted that staff routinely operate on (potentially false) assumptions derived from past experience, cultural knowledge, and expectations. This research also emphasised the differences and potential for conflict between how systems and people work, noting that providers remember patients by their conditions rather than name or other formal identifiers used by systems. One study looked specifically at the issue of uncertainty for primary care providers, in terms of how they prioritise when faced with competing demands and time pressures, and how they diagnose and manage patients [72]. Providers use a combination of “book knowledge” and “experience-based knowledge,” and apply heuristics to patient care. They assume “horses first, rather than zebras” but also admit to uncertainty and “reserve space for re-evaluating their judgements”. As such, the author did not believe uncertainty was “necessarily detrimental to safety” but rather that it, unlike standardisation, enabled providers to use their expertise and develop “original responses” to a given situation [72].

### Subset 5: Primary / Secondary Care Interface

#### Overview

Nine papers were focused on issues at the interface of primary and secondary care. Most were concerned with discharge or so-called ‘handover’ or ‘handoff’ or processes between hospital departments and general practice, although one was concerned with issues in continuity of care more generally [75]. Following appraisal, all papers were considered acceptable by the reviewers in terms of reporting of research methods except for one which was considered “excellent” [76] and two on the borderline of excellent [75,77]. Compared with other subsets of articles there was a very wide geographical spread in terms of study location with two studies having been completed in the USA [76,78]; one in the UK [75], one in Spain [79], one in Switzerland [80], one in Sweden [81] and one in the Netherlands [82]. Three of these articles involved country-specific reports that formed part of the pan-European “HANDOVER” study [79,81,82], the main results of which were considered in two articles reporting the overall results from The Netherlands, Spain, Poland, Italy and Sweden combined [77,83].

The participants in the seven country-specific studies or sub-studies included patients only [75,78,81], professionals only [76,80] or a combination of both [79,82]. Five studies or sub-studies were concerned with specific groups of patients: those with chronic diseases [81,83], multiple long-term conditions [75] or patients who were vulnerable [79] or elderly [78]. The latter study stood out as having a sample consisting of 69% females and 70% African Americans. It was also mixed-methods in approach, although qualitative data were collected in telephone interviews [78]. The remaining studies all relied on semi-structured interviews apart from one that utilised focus groups [80] and one that used a combination of semi-structure interviews and focus groups [77].

#### Findings

The findings translated according to the concepts “resources,” “constraints,” “role” or “agency” and “effectiveness” or “efficiency” with reference to patients, staff or health care systems (Table 6). A small group of findings, labelled “external environment” were concerned with patients’ home social or economic circumstances. Because the findings were concerned with issues in both primary and secondary care, some of the hospital specific issues (e.g. “a positive bedside manner”) have been removed from Table 6 for the purposes of the analysis presented here.

**Table 6:** Translation table—Findings of studies about the primary/secondary care interface.

	Resources	Constraints	Role / Agency	Effectiveness / Efficiency
Patients	“not properly prepared for discharge” [78] [77]	“especially the elderly, often are not aware of the importance of the information provided, unable to remember the information and overwhelmed when they are suddenly told they have to leave the hospital” [77]	“in the hospital, you just have to surrender yourself” [79]	“the importance of patients in contributing to an effective handover” [83]
	“with greater health literacy and language skills would be more likely to navigate the health system safely” [79]; “patient-specific resources, self-management capabilities and skills are often overestimated or not critically assessed” [77]	“Patients with limited personal resources or with low health literacy had difficulties understanding the received information and sharing” it [83]; “patients with language barriers and low health literacy may be unable to provide full information” [79]	“they could not participate actively in follow-up” [83]; “having difficulty” or “delay” “obtaining follow-up tests or appointments” [78]	“when they assumed responsibility for the handover, communication worked better. It also empowered them and gave them a sense of control over the handover process” [83]
	“discharged with unclear or insufficient information on how best to handle their medicat-ions” [83]; “[no] proper discharge materials which then caused other issues”[78]; discharged “without detailed instr-uctions … for how to perform simple proced-ures such as changing a wound dressing”; with-out sufficient medicate-ons or other supplies”; “insufficient instruct-tions concerning their follow-up” [77]; “Information can be an important resource for patients, especially in the context of multiple morbidity” [75]	Patients who knew that information was transferred electronically “did not consider it necessary to participate in the handover communication between settings” [81]	“perceived their active involvement was required for an effective handover” [83]	Patient data transfer not “safe due to the conflicting information it can produce, and to the unreliable nature of the transfer process” [79]
		“vulnerable patients are at particular risk of experiencing breakdowns in communication during the handover” [79]	“often were not informed about the nature of the information being transferred” [79]; “a smoother information flow without them acting as the conduit” [79]	“sudden and abrupt discharge that overwhelmed patients” [77]
			“guided by their past experiences, which taught them how to interact and communicate effectively with healthcare providers” [81]	
			“The role and responsibility of patients in their handover is not clear to the healthcare professionals involved” [82]	
			“having appointments made for them and the degree of flexibility over appointments was a concern” [75]	
Staff	“Most GPs were quite flexible about the method of communication” [80]	“a negative climate for communication involved healthcare professionals neglecting patients’ individual needs, or being too busy to communicate” [83]; “Giving priority to delivering medical or nursing care” [77]	“Patients assumed handovers are performed by healthcare professionals” [83]	“healthcare providers responding to the patients’ handover information, indicating their understanding of the patient’s situation and adjusting their communicated information to patients’ needs and abilities” [81]; “more effective when healthcare professionals were actively involved”[83]; Report from hospital nursing staff “was often missing, incomplete or not provided in a timely manner” [79]
	“Provider-to-provider handoff … may also facilitate patient-centeredness because providers exchange information between visits, which enables next providers to “jump start” subsequent visits by demonstrating specific knowledge of a patient’s experiences, medical history, and symptoms”; “curbside consultation … a brief, face-to-face conversation between providers about professional matters when they opportunistically encountered one another in a private hallway or other area” [76]	“when patients felt a negative or indifferent attitude it influenced their participation”; “decisions that appeared to be made against explicit patients’ wishes or without their knowledge”; “They would limit information shared with healthcare providers if they distrusted or felt uncomfortable with them … patients would wait to give or ask for information until a specific trusted person was available” [81]	“transfer nurse” [83]	“Because the patient is not present, providers are free to use technical terminology … which may contribute to clinic efficiency”; “The temporal immediacy of the provider-to-provider exchange”; “patients benefit when a second provider demonstrates specific knowledge of a patient’s experiences, history, and associated symptoms” [76]
	“Patients wanted to communicate with personnel they knew and/or had good experiences in past dealing with” [81]	“healthcare professionals characterised the patient’s role in the discharge handover as limited to a passive conduit function … Patients did not perceive this role as positive” [79]; “healthcare providers did not facilitate patients’ contribution to handover communication, asking few questions during the handover” [81]	“often discharged with a medication different to that taken before admission … not always clearly communicated to their” GP [79]	
			“Patients perceived a positive climate for communication, based on mutual respect, in an open atmosphere and on a personal level, between them and the healthcare profession-als as an enabling factor for participation [handover]” [83]	
			“Whether … the GP treats the patient as a whole person, shows an ability to listen, takes time to explain things in a kind manner” [75]	
			“many patients expressed the feeling that their physicians were obligated to communic-ate with each other” [78]; “Patients desire appropriate communications from health care professionals” [75]	
			“important that community care providers check with patients whether there are unresolved issues to be dealt with … this frequently does not happen” [77]	
External Environment	“Managing a handover for a patient with a difficult or unstable social situation or with limited cognitive abilities is more demanding for doctors and nurses” [79]	“Patients and their family members are expected to take on significant responsib-ilities in the handover process, as well as handling administrative issues related to treatment such as filling prescriptions and managing home care”; “patient referral information is often limited and frequently does not include information on nursing requirements or the socio-economic situation at home” [79]	“GPs stated that during the hospital stay they are often confronted with patients’ relatives, who also seek information” [80]	
System	Shared EHRs “a possible solution to address handover problems” [79]	“problems with written referral… emerg-ency admission is usually managed on the phone”; delayed discharge papers “a major issue” [80]; “lack of personal contact [between staff]; “potential to miss out on crucial information” [79]; inform-ation about “patients’ social and emot-ional status … often is not present or deficient” [77] (similar in [79])	“it is not how communication takes place that is important, but the fact that it takes place at all” [80]	“GPs stated that they saw large hospitals … as a kind of a “black box”; “importance of a minimum standard of communication” [80]
	“GPs mostly working with paper-based records” [80]	“organizational barriers to relational con-tinuity”; “difficulty of making appoint-ments to see the same professional”; “appointments and responses to urgent requests” [75]; “sends the letters to adm-inistration who print it … increases turn-around time”; “variety of systems, inad-equate training on their use, lack of IT support personnel … extra time needed to complete the electronic forms” [82]	“Longitudinal continuity is valued by patients because it facilitates the establishment of a shared personal and clinical history between two individuals, rather than each visit constituting an unconnected encounter between relative strangers” [75]	“not … an effective solution, as not all healthcare providers have access or contribute data to such systems” [79]
			EHRs “not currently used in the context of inter-organisational handovers” [79]	“Patients perceived a gap between the information they received and … they … needed for cont-inuous care [especially about] medication” [83]
			“Electronic handoff” … facilitates contact among and between providers to transfer patients with routine, non-urgent problems” [76]	“GPs are not always up-to-date with the treatment that was provided and the follow-up that was advised during the hospitalisation” [77]
			“Patients were often uncertain of whether and how communication between the inpatient physician and PCP took place” [78]	“miscommunication between sites” [75]
		“negatively influence patient engagement because the process may take place o**ver** a period of weeks or” months [76]; “pat-ients expressed the need for a dedicated discharge encounter” [83]; no “standard discharge consultation” [77]; “lack of formal handover encounters” [81]	“visits to secondary care were often sufficiently infrequent that they were inexperienced in manag-ing these difficulties and … required [more] assist-ance from staff” [75]	
		“shift work structures”; “lack of time”; “pressure on available hospital beds”; “discharges on weekends” [77] (similar in [75]); “When … perceived as stressful and time constrained, patients neither rec-eived nor gave as much information” [81]		

Although the quality of the studies was high (see above), the translation of findings did not seem to lead to concept development, perhaps because they were mainly concerned with highlighting ‘factual’ issues in problems in hospital handover or handoff. Only one study used a conceptual model [75] and only one was explicitly concerned with generating theory [76].

The findings concerned with patient resources mostly pointed to the fact that patients had often been discharged from hospitals without adequate preparation, information and materials, or supplies (e.g. of dressings or medicines). Constraining factors mainly pointed to the vulnerable characteristics of certain patient groups in terms of their ability to understand or communicate information. In considering the effectiveness of ‘active’ patients in the handover process, refutational findings were evident. Thus, whilst one study noted that “the importance of patients in contributing to an effective handover” and that “communication worked better” when patients “assumed responsibility for the handover” [83], another noted “a smoother information flow without [patients] acting as the conduit” [79]. On the face of it, this would seem to suggest that patients might assist with improved communication (with health professionals) but are not an effective way of exchanging information between health professionals. In considering the agency of patients in the handover process, the findings seemed to underline that whilst ‘active’ patients might be preferable in handover terms, this role is not one that is generally facilitated by staff or health system: “in the hospital, you just have to surrender yourself” [79].

The findings in relation to medical staff underlined the importance of good inter-personal relationships between staff and the benefits of person-person to communication, even if such encounters were brief. Roles and constraints echoed the findings found in the subset of studies concerned with patients’ perceptions with an importance attached to staff being receptive to patients’ wishes and needs and imbuing patients with a sense of trust and feeling valued. Findings concerning inter-personal relationships between staff were echoed in those concerned with systems issues with a pre-eminence once again being attached to communication: “it is not how communication takes place that is important, but the fact that it takes place at all” [80]. Also following from findings concerning staff, it was suggested that telephone communication between professionals (being one-to-one communication) is preferable to written letters [80]. As seen in the subset of studies focused on patients’ perceptions of safety, opacity of systems emerged as an important concept as patients were “often uncertain of whether or how communication” between care providers had taken place [78]. Similar issues were reported by GPs in one study whereby large hospitals were viewed as “kind of a ‘black box’” in systems terms [61].

## Discussion

### Synthesis: Blame the patient, blame the doctor or blame the system?

In the introduction, it was noted that previous synthetic and other studies of patient safety in primary care have been most concerned with developing classifications, lists or taxonomies or errors and harms. That is, they sought to describe what kinds of errors or avoidable harms occur in primary care. Although this approach was also evident in some of the studies examined here, a synthesis of the findings of qualitative studies of patient safety in primary care showed that they were rather concerned with why errors occur. The bulk of these findings broke down into explanations that lay in the behaviour or characteristics of patients and health care staff, or in organisational or systemic factors. To put it simply, findings seemed either to “blame the patient,” “blame the doctor” or “blame the system.” The findings were thus fundamentally about the lack of ‘fit’ between infallible and “variable” human beings and perceived overly rigid guidelines, procedures and computer systems. Other challenges derived from the autonomous nature of medical power coupled with the kinds of time, resource and workload issues generally characteristic of family practice settings.

In Tables 7–11, an attempt has been to characterise the raw findings contained in Tables 2–6 in reduced manner that effectively captures the major content of each cell of the original tables. On occasion, this has led to some conceptual development. For example, the characteristics and behaviour of patients seen to adversely affect safety (Table 2) have been captured by the synthetic metaphors “physical and cognitive disadvantages” and “wrong behaviour” (in Table 7). Similarly, the behaviours of staff seen as adversely affecting safety (Table 2) have been represented by the metaphors “arrogance” and “incompetence” which seem to best capture and encompass the kinds of “prejudice” [35] “inattention” [39] and inadequate training [35] found in the primary studies. In other cases, findings, concepts or metaphors used by the authors of the primary studies seemed to already best capture or represent the main issues. In these cases, reference to the original studies has been retained in Tables 7–11. This seemed to happen more often in the concept reduction and development tables concerned with medication safety (Table 9) and organisational or systems issues (Table 10).

**Table 7:** Concepts and metaphors in studies of patient perceptions of safety in primary care.

	Patients	Staff	Doctor-Patient Communication	System
Adversely Affects Safety	Physical and cognitive disadvantages	Arrogance	Unsuccessful, insufficient or unclear communication	Failures of access
	“wrong” behaviour	Incompetence		Lack of service availability due to resource constraints
				Dispersed patient information
Unsafe Practice	Negative emotional responses	Arrogance leading to mistrust	Magnification of insults within relationship breakdown	Mistrust in unfamiliar providers
Actions to Promote Safety	Active stance	Making patients feeling respected, valued and able to speak up	Open communication	Keeping patients informed
	Skilled self-managers; Informed; Adaptable in response to system			
	Use social networks		Use of patient advocates	

**Table 8:** Concepts and metaphors in studies of GPs’ perspectives.

	Threats to Safety	Reducing Error
Primary Care staff characteristics	Clash with personal life	Neglect personal life
	Lack of training	Get training
Primary care staff behaviour	Usual clinical practice; Not keeping a cool head and emotionally detached; Not seeing the wood for the trees	Trust one’s instincts; Get training; Share knowledge and training; Learn from mistakes; “Deal with uncertainty” [34]
Patients characteristics	Complex symptomology; Social circumstances	
Patient Behaviour	Wrong behaviour	Self-responsibility
Doctor-Patient Communication	Lack of communication; Competing agendas; GPs jumping to conclusions	Training in doctor-patient communication; Improved medical records
Organisation of Care	Issues out of the GP’s hands; Lack of communication between primary and secondary care; Work overload within resource and budgetary constraints; Absence of or ignorance about policies, protocols and guidelines; Alert fatigue; Redundant, cumbersome or inadequate computer systems	“In an ideal world …”; Effective communication between primary and secondary care; Teamwork; Better resources
Professional roles	Deficiencies in medical education; Relative professional status of nursing staff; Clinical autonomy	Double checking; Reliable systems; Safety learning culture; Teamwork

**Table 9:** Concepts and metaphors in studies of medication safety.

	Social	Technical
Characteristics of patients	Problematic presentation; Cognitive and educational deficiencies	Risks vs. Benefits of taking medications; Mismatch between patient behaviour and system requirements
Relationships between primary health care staff	Deference towards GPs; Clinical autonomy creates communication problems; A need for “free” and open two-way communication without fear of blame	Pharmacists’ access to GPs and medical records; Poorly performing GPs; The degree of face-to-face contact between different health workers
Communication between patients and staff	“To create a feeling of safety” [63]; Communication between doctors and patients mediated by receptionists and telephones (potential for further errors)	Pressures of time; Prescribing reduces face-to-face contact between patients and staff
Knowledge	Insufficient time for medication counselling with patients; Inflexibility and irrelevance of guidelines; New drugs	Lack of transparency and access to information in EHRs; Drug and therapeutics training “picked up on the job” [59]
Responsibility	Whosoever has the responsibility gets the blame; Responsibility and control resides with the patients’ own doctor or prescriber, but it needs to be shared out with other doctors and patients; A tension between getting patients involved and eroding professional trust (and power?)	Power versus competence; Systems versus ad hoc approaches
Workflow	Getting around unhelpful guidelines and systems within pressures of workload	Time and resource constraints mean adhering to guidelines or systems can introduce ‘new’ errors; Working around and dealing with unhelpful computer systems

**Table 10:** Concepts and metaphors in studies focussed on organisational and systems issues.

	Threat to Safety	Reduces Error
Characteristics	Byzantine organizational structures; Dilemmas of managing trade-offs between different risks within time and resource constraints	“Surveillance and performance management” [73]
Functionality	Electronic medical notes brought new errors and needed workarounds; a further drain on precious time and resources; Variability in patients’ and staff abilities	Timely accessibility and updatability of information; “involving patients can act as an additional check to prevent serious errors” [68]
Assumptions, Illusions and Uncertainties	Organizational Reputation [73]; “technology … does not support the cognitive work of healthcare staff” [70]	Fitting the organization to patients’ needs and expectations within a culture promoting safety; Learning to deal with uncertainty; Learning from prior experience
Standardisation	Lack of fit between protocols and systems on the one hand and “dynamic” [70] humans on the other	Standardization and improvements in knowledge, regulations, reporting and processes

**Table 11:** Concepts and metaphors in studies of the primary/secondary care Interface.

	Resources	Constraints	Role / Agency	Effectiveness / Efficiency
Patients	Lack of preparation, health literacy, self-management skills and information ahead of hospital discharge	Deficiencies in patients’ cognitive and other abilities	Active versus passive role unclear to patients and professionals; partly dependent upon prior experience	An active role led to a more effective handover, but not a reliable means of data transfer
Staff	A flexible approach to direct provider-provider communication	Not prioritising patients’ needs for direct communication affected trust and subsequent encounters; Patients as passive during handovers	Care and drugs not transferable between primary and secondary care; Respect and trust for positive communication; Importance of provider-to-provider and providers-patient communication	Staff understanding patients’ circumstances, clinical history and needs
External Environment	Need to involve patients’ relatives			
System	Non-compatible records systems in primary and secondary care	Importance and value of direct communication between GPs and hospital staff; Conflicts with resource issues and staff time	“Longitudinal continuity” [75]; Opacity and utility of electronic methods of handover?	System workaround; Information gaps; Mis-communication

Issues of patients’ characteristics and behaviour were represented in all of the article subsets, but were least apparent in that concerned with organisation or systems issues (Table 10), likely reflecting that patients have little input or impact themselves on the organisation of care. Findings from all article groups referred to the complex symptomology and physical or cognitive disadvantages found, especially in elderly or multimorbid patients. These issues were probably best captured by the third order interpretation “problematic presentation” on the part of patients and/or “problematic attitudes” on the part of staff as derived from the medications studies. Other important themes related to the usefulness of patients adopting an ‘active’ stance in relation to healthcare encounters, which was especially evident in the findings of studies conducted at the primary/secondary care interface (“patients” row and “effectiveness and efficiency” column), and in the “reduces error” column of the organisations and systems table (Table 11). However, isolated studies in the medications [66] and primary/secondary care interface subsets also pointed to the ways in which relying on patients (e.g. for information transfer) [79] might rather compromise their safety.

From the patients’ perceptions studies, staff behaviour seen to adversely affect safety involved various types of arrogance or incompetence where perceived insults could have consequences beyond the immediate situation, influencing future clinical relationships and help-seeking behaviour. Similar findings derived from the hospital-side of the primary/secondary care interface studies, which makes clear that such issues are not unique to primary care.

Systems issues were evident in every group of studies. For both staff and patients, the main threats to safety seemed to derive from busy physicians’ offices that were operating under budgetary, time and resource constraints. For patients, the main issues concerned lack of timely access to services and dispersed medical information. For primary care workers (Table 8), the main issues revolved around workload, teamwork, a lack of communication with secondary care and redundant, dysfunctional or inadequate computer systems. Although deficiencies in medical education and training were mentioned across the studies, issues of clinical autonomy and the relative professional status of nursing staff or pharmacists were only mentioned in the staff and medications studies.

**Fig 2:**
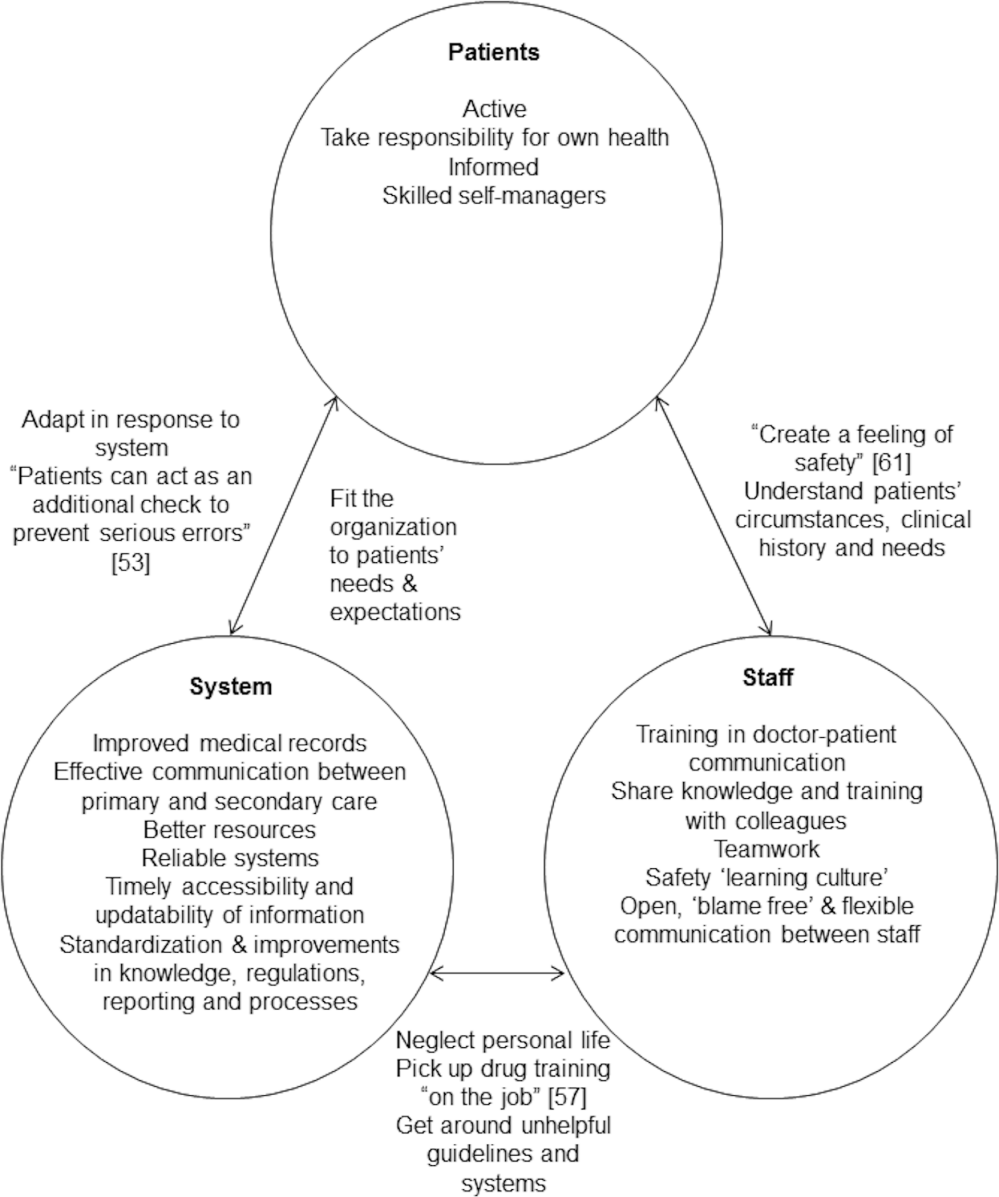
Promoting Patient Safety in Primary Care.

**Fig 3:**
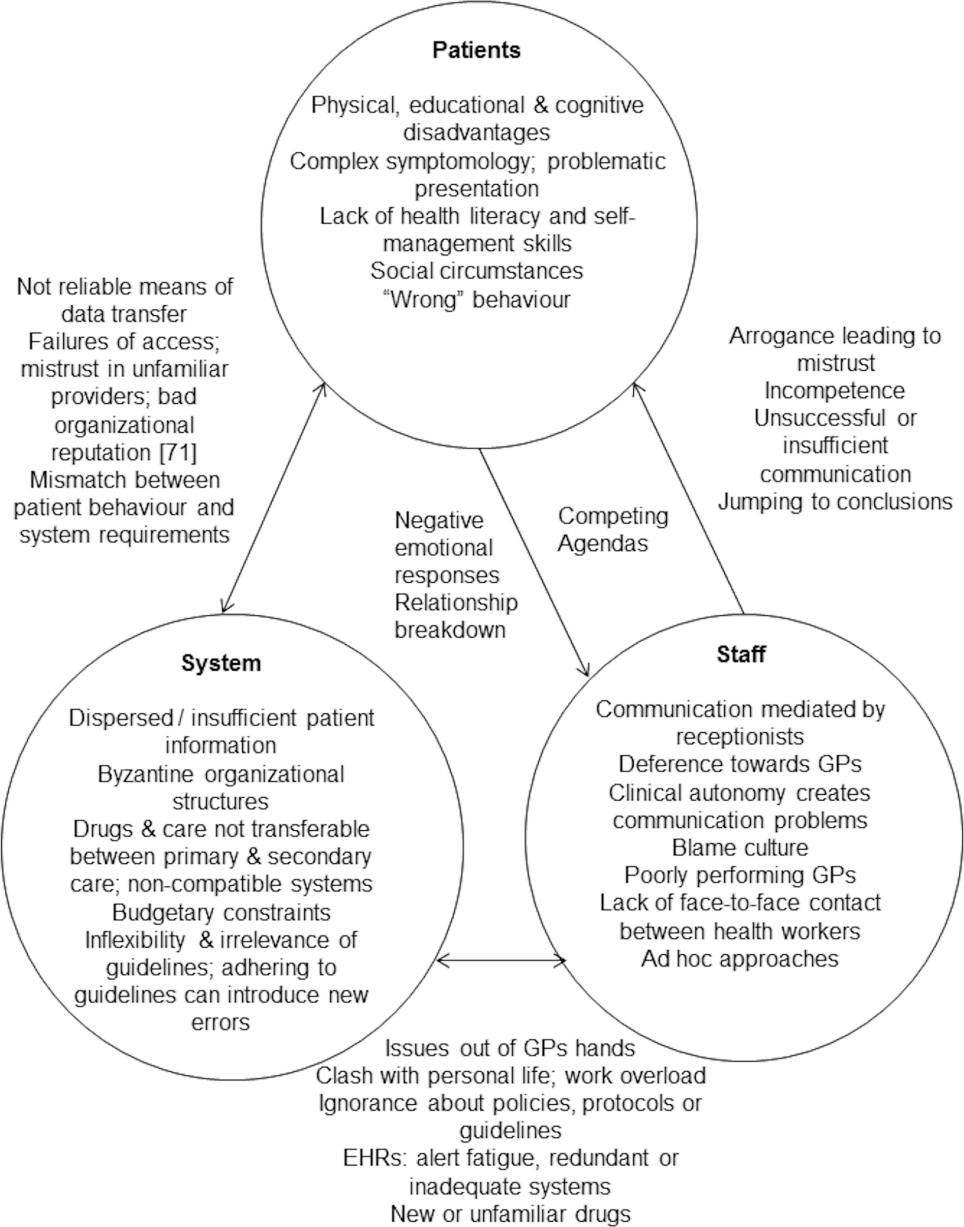
Degrading Patient Safety in Primary Care.

Synthesizing the translations from the different subsets of studies (showed that they were principally concerned with the factors, processes or issues that either promote (Fig 2) or degrade (Fig 3) patient safety in primary care. The main issues grouped into the characteristics or behaviour of patients or staff; interaction between patients and staff or staff and other staff; and organisational or systems issues that confronted patients or staff. Many issues point to the fact that human beings do not always “fit” systems or behave or perform in ways that they are “supposed” to. The main threats seem to centre on time and resource constraints. Simply, if family practices had more staff, more resources and more time to spend with each patient, then they would be safer places for patients. Other issues point to the historic power and autonomy of doctors which again presents challenges for “standardised” systems or operating procedures.

As was noted in the introduction, EHRs are not a panacea, and the assumption that electronic records are inherently “safer” than paper ones might itself diminish safety if their use reduces doctor-patient communication or offers an illusion of safety leading to “hidden hazards” [34]. An important tension was evident in the findings concerning the use of guidelines or protocols. On the one hand, they are regularly put forward as a means of improving safety through standardisation and dissemination of good practice. On the other hand, if consultations are done by rote then important information coming from the patient may be missed, especially if the physician’s attention is focused on a computer screen [16]. In the UK, the implementation of a “Quality and Outcomes Framework” (QOF) means that primary care consultations are becoming reduced to a standardised set of questions and procedures which might be completely at odds with the reasons why a patient is visiting their GP in the first place [84]. This is particularly important given the constraints on consultation time, for in the context of a ten minute consultation whether or not a GP focuses on the needs of the patient or on the “needs” of activity that is financially incentivised through the QOF could affect patient safety outcomes (in either direction). Further, some GPs have questioned whether guidelines are relevant for those groups of patients who appear at increased risk of safety failures. Thus, whether “*ad hoc*” [60] or “gut” instinct [50] approaches are in fact safer than following a guideline is an interesting issue that would be worthy of further study by patient safety researchers. This might reflect an issue in translating hospital-based safety research or initiatives into family practice settings where a high degree of uncertainty and a “let’s wait and see” approach [72] are part and parcel of everyday practice. Alternatively, it could be that protocols or guidelines are good for some things (e.g. communication of test results) in primary care, but not for others.

### The moral and emotional foundations of patient safety in primary care

A conceptual interpretation of the findings points fundamentally towards patient safety as an organizational moral framework for evaluating people’s feelings about health services. A common narrative found in the studies concerned the difficulties of fitting the emotional and relational aspects of safety within “the system” of health care, which appeared perceived in (bureaucratic) “machine” terms [85]. Health workers act as the interface between patient and machine which is why communication with them is of fundamental importance to safety. If information needed by the machine fails to leave the patient, or is written off by the recipient, then the consequences could lead to harm. This harm could well be of long latency, for example if it causes a breakdown of relationship trust which results in a different (or more serious) problem further down the line. Harms could further result if essential information fails to be communicated by a clinician, such as the disclosure of a diagnosis or advice on condition self-management [86]. In a recent editorial published alongside a collection of patient safety articles in a different journal, it was noted that, “there are unresolved ontological debates, for example, about whether patient safety is a tangible ‘thing’ that can be precisely defined and elaborated as a taxonomy of events, or whether it remains a more nebulous, contested and culturally relative concept”[87].

Both aspects were clearly evident in the studies reviewed and synthesized here, with most of the findings focused either on perceived threats to safety or the nature of potentially preventable adverse events. The main threats to safety concerned failures to communicate or transfer medical information and a panoply of systems issues that derived basically from the fact that general practices are busy settings with finite resources. Systems of health care appear as highly demanding in terms of patient and staff inputs (of all kinds) and a fundamental issue concerns the pros and cons of standardisation involving protocols, training, and patient and staff presentation and behaviour. These findings denote highly internally socialised judgements and assessments about what it is realistic or reasonable to expect in clinical interactions. Judgements about the appropriateness and ‘safety’ of interactions or systems boil down to moral judgements about patients (“wrong behaviour”), staff (“incompetence,” inappropriate attitudes) or systems (principally through failures of access or provision). The perceived shortfall in primary care systems present instead perhaps as a series of moral imperatives (busy settings; redundant, dysfunctional or inadequate computer systems; disadvantaged patients; overworked staff and a well-recognized mismatch between systems and people).

Issues around trust in health workers or organizations were articulated in several primary studies and are most evident in our own interpretations concerning the perceived “opacity” of clinical processes or systems; the potential for a “blind faith” in EHRs to compromise safety; and other “assumptions, illusions and uncertainties” that characterize the “byzantine” structures of health care management and the messy and uncertain nature of clinical work. Interpretations found in two studies (“reality gaps” [58] and “hidden work” [63]) pointed to the potentially “nebulous” [87] nature of patient safety, as something as yet unknown, concealed or waiting in the wings. According to a recently published handover study that was not included in this review, “The threats to safety are located between care providers, processes and settings” [88].

This meta-synthesis has adopted a narrative approach to synthesis built on concepts and metaphors extracted from the included studies using elements of “line of argument” (as above) and “reciprocal translations” [21] (as in Tables 2–10). Conducting a meta-ethnography can lead to conceptual development but does not always do so [89]. The conceptual development here was limited. The main contribution to knowledge concerning safety as “a tangible ‘thing’” [87] came through refutational groups of findings, for example concerning the role of patient agency in safety or the seemingly equivocal effects of EHRs, protocols and guidelines on engendering a feeling of safety. A conceptual reading of the studies, developed as a line of argument above, points to safety as somehow elusive—perhaps because it is a subjective feeling, drawing on moral perspectives and with sometimes unseen psychological consequences. Another reading of the studies suggests that patient safety failures are inevitable given the way that health services are set-up, organized and managed. These findings support recent calls for a focus on relationships in patient safety research [87]. They also suggest that a more effective way to improve safety in primary care might be to focus on interventions that operate at the systemic or organizational level and involve changes to funding allocations, contracts, working patterns, targets and medical training rather than those focused on behaviour within individual services.

### Methodological Considerations

As demanded by the journal editors, a PRISMA flow chart was constructed to illustrate the results of literature searches and the exercise of inclusion/exclusion criteria. However, this was not found to be useful in reporting a meta-ethnography. The PRISMA guidelines were drawn up by an expert panel of clinicians and statisticians and are aimed principally at reviews or meta-analyses of trials or other evaluations of interventions [90]. Effectiveness reviews start with an *a priori* research question and the review follows in linear fashion. However, a meta-ethnography is concerned with what a body of literature ‘says,’ and the research question and inclusion criteria may be subject to change through the different stages of the review or meta-synthesis. For example, in this study it was not clear that findings concerning attitudes to error reporting did not fit until repeated attempts were made to translate findings into one another.

A subsidiary aim of this study was to investigate means of searching for and identifying qualitative studies for a systematic review. As was noted in the introduction, identifying every single relevant study may not be critical for a qualitative synthesis [25] and meta-ethnographies usually involve small numbers of primary studies. Too much time spent searching could even be detrimental given trade-offs in relation to the time available for other higher level activities such as conceptual development [29]. In relation to systematic reviews of quantitative studies, one report suggested that the coverage of Google was “100%” for available primary studies [91]. However, as has been noted by others, what is in Google Scholar and whether it will appear in searches or not are two different issues, as, “Looking for papers when you know their titles is a far different issue from discovering them initially” [92].

In this study, we were interested in what would be found by different means by using a simple search that could be replicated across different databases and platforms. We did not search for papers that we already knew about and we used blinded reviewers who were not aware what each had discovered until the exercise was completed. Use of a simple search limited the full features of specialist bibliographic databases. Whilst such a simple search appeared to suggest that Google Scholar was able to find more relevant materials, given that it was only possible to assess the first 1,000 results (of 17,200 returned), the number of “hits” found beyond 1,000 was useless. Overall, our results reasserted the importance of searching by different methods, including hand searching, reference list searching and searches of specialist databases, as each method produced unique results that were not obtained by other means. Having said all of that, the yield from Google Scholar was greater than that from other sources which points to a need for further research.

### Strengths and weaknesses

We used a simple search strategy so as to allow blinded comparison of Google Scholar and specialist bibliographic databases in order to contribute to development of methods of searching for studies. However, a simple search that could be used in both platforms was probably not optimised for either. A particular problem with Google Scholar is that the searching algorithms are not made public [92]. The limited number of search terms and strings employed may have limited the number of relevant articles found, although other sources of searching were also used. For example, the word “interview” was not included. However, in this specific case, pilot searches and checks undertaken during the manuscript review process both showed that this particular omission had little impact on the number of included studies, although it did increase the number of database “hits.” It is not clear whether an interpretive synthesis of qualitative studies needs to find every relevant article, nor whether the addition of further studies would have wildly affected the results (James Thomas and Andrew Booth, personal communications). Inclusion of further studies may have been unmanageable. The study was undertaken by a large team of experienced qualitative researchers, which is essential in meta-ethnography [25]. However, despite a mechanistic approach to certain aspects of the synthesis, meta-ethnography remains a subjective enterprise and other workers from different backgrounds or disciplines may have highlighted other findings than those that we did. The main weakness of our approach is that it was heavily descriptive. Whilst reporting standards for meta-ethnography have not yet been developed [87], our experience is that the results of a meta-ethnography are dependent more on the skills and prior learning of the synthesizer than they would be on fidelity as determined by a checklist of tasks around searching, appraisal, data extraction, translation and synthesis.

## Conclusions

Previous studies of patient safety in primary care have identified the types of medical errors or patient safety issues found in primary health care. These have typically been related to knowledge gaps in health care practitioners [7] or “process errors” arising from communicative, clinical or administrative factors [6,8]. Factors related to communication, skills and “office processes” [8] were also highlighted in this meta-synthesis of qualitative studies of patient safety in primary health care. However, the characteristics and behaviour of patients themselves was also underlined as a significant threat to patient safety. It is known that patients’ definitions of mistakes tend to be broader than clinical issues and encompass violations of trust or criticisms of the stance or attitudes of medical practitioners [12]. The results of this synthesis also assert the possibility or necessity for an active, informed or “involved” patient in order to reduce threats to patient safety [93]. However, a hospital-based interview study has highlighted the possible negative consequences that can follow from increased patient involvement in their own care, which in some circumstances can also lead to reduction in trust or problems in the relationship between patients and health care professionals [94]. Similar issues arose in one of the community pharmacy studies included in this meta-synthesis [66].

On the face of it, very serious questions remain about what can be achieved in the context of the increasing demands being placed on primary health care globally which continue to impact upon the time and resources needed to keep systems safe and promote a culture and feeling of trust for patients. The results of the synthesis add to previous primary studies that point to the hidden costs or unintended consequences of increasingly routinized and technological processes, such as EHRs [16], e-Prescribing [17,18], clinical guidelines and protocols. One of the basic findings of this meta-synthesis is that safety for patients is predicated upon direct face-to-face communication between them and health workers; and that similar direct communication should occur between different health workers themselves. Currently, patients and staff appear to face twin threats of constraints of time and resources (e.g. difficulties getting appointments and limited consultation time) and increasing technicalization which reduces the capacity or opportunities for face-to-face communication. The importance of being treated with respect and dignity was mentioned by patients in qualitative studies of patient safety, but such matters are not generally considered as safety issues in the hospital focused literature on patient safety which has tended to focus on systems approaches, such as automation of tasks [95]. However the industrialization of care processes and the commodification of patients (e.g. as sources of remuneration in incentivised managerial processes such as the QOF in the UK) has great capacity to threaten the more human aspects of the clinical encounter in primary care. Another key issue relates to the autonomous nature of clinical practice and the ‘autonomous’ nature of patients who vary in their skills, abilities and engagement with health care and condition management. However, at base, issues of human autonomy would once again seem to point to a fundamental clash between standardized systems on the one hand and ‘unstandardized’ individuals on the other.

One of the main concepts that derived from the subset of studies concerned with patients’ views of patient safety in primary care was the “opacity” of systems. That is, patients were often ignorant of usual practice or standard operating procedures (SOPs), e.g. with regard to notification of test results [41]. However, similar issues in relation to the infallibility of SOPs were also evident in the subset of studies focused on staff perspectives, with GPs often seen to prefer to “do their own thing” [46] rather than follow guidelines or protocols. Further research is needed into the contexts and circumstances in which protocols or guidelines are “safe” in primary care, and those in which they are not. Another key issue for GPs related to work/life balance, with an assumption that GPs needed to work long hours in order to provide “safe” care, given the resource constraints alluded to above. It should be self-evident that a “safety system” which relies on people overworking and neglecting other aspects of their lives is doomed to failure. Clear practical issues for improvement were identified, mainly involving team work and training, which are perhaps unsurprising. Another key issue, where findings appeared sometimes refutational, was the extent to which GPs should take patients’ concerns at face value. On the one hand, ignoring what a patient is saying is clearly a risk in safety terms. On the other, if a patient lacks understanding of their own health needs or management issues, paying too much attention might also constitute a risky activity. Again, this seems fundamentally to point to the variability, individuality and unpredictable nature of clinical presentation which is one of the key challenges (and interesting facets) of clinical work in primary care.

## Supporting Information

S1 ENTREQ Checklist (DOCX)

S1 Appendix (DOCX)
