# Supplementary material for: Blame the Patient, Blame the Doctor or Blame the System? A Meta-Synthesis of Qualitative Studies of Patient Safety in Primary Care
Source: PLoS One. 2015 Aug 5;10(8):e0128329. doi: 10.1371/journal.pone.0128329 (PMC4526558; doi:10.1371/journal.pone.0128329)
Supplement: S1 ENTREQ Checklist — (DOCX) [file pone.0128329.s001.docx]

**Tong A, Flemming K, McInnes E, Oliver S, Craig J. Enhancing transparency in reporting the synthesis of qualitative research: ENTREQ. BMC Med Res Methodol. 2012;12(1):181.**

| **No** | **Item** | **Guide and description** | **Response (Page No. in manuscript)** |
| --- | --- | --- | --- |
| **1** | Aim | State the research question the synthesis addresses. | To develop a conceptual model of patient safety in primary care (abstract, p.1; introduction, p.5) |
| **2** | Synthesis methodology | Identify the synthesis methodology or theoretical framework which underpins the synthesis, and describe the rationale for choice of methodology *(e.g. meta-ethnography, thematic synthesis, critical interpretive synthesis, grounded theory synthesis, realist synthesis, meta-aggregation, meta-study, framework synthesis).* | “Meta-ethnography”: Abstract, p.2; Materials and Methods, p.6) |
| **3** | Approach to searching | Indicate whether the search was pre-planned (*comprehensive search strategies to seek all available studies)* or iterative (*to seek all available concepts until they theoretical saturation is achieved)*. | Pre-planned (Methods, p.8) |
| **4** | Inclusion criteria | Specify the inclusion/exclusion criteria *(e.g. in terms of population, language, year limits, type of publication, study type).* | See Methods, pp. 7-8 |
| **5** | Data sources | Describe the information sources used (e.g. *electronic databases (MEDLINE, EMBASE, CINAHL, psycINFO, Econlit), grey literature databases (digital thesis, policy reports), relevant organisational websites, experts, information specialists, generic web searches (Google Scholar) hand searching, reference lists)* and when the searches conducted; provide the rationale for using the data sources. | See “Search strategy,” p.8 |
| **6** | Electronic Search strategy | Describe the literature search *(e.g. provide electronic search strategies with population terms, clinical or health topic terms, experiential or social phenomena related terms, filters for qualitative research, and search limits)*. | A simple search was used (Reproduced in Box 1, p. 51) |
| **7** | Study screening methods | Describe the process of study screening and sifting *(e.g. title, abstract and full text review, number of independent reviewers who screened studies).* | Initial sifting was done by title and abstract review in team meeting (table 1, p.52) |
| **8** | Study characteristics | Present the characteristics of the included studies *(e.g. year of publication, country, population, number of participants, data collection, methodology, analysis, research questions).* | This information is included in narrative form for each subset of studies, including (at minimum) country, data collection methods, populations and focus/research question (pp. 13-14, pp. 17-19, pp.22-23, p.28 & pp.32-3) |
| **9** | Study selection results | Identify the number of studies screened and provide reasons for study exclusion *(e,g, for comprehensive searching, provide numbers of studies screened and reasons for exclusion indicated in a figure/flowchart; for iterative searching describe reasons for study exclusion and inclusion based on modifications t the research question and/or contribution to theory development).* | Exclusion was iterative, as it depended in part on the ongoing bounds of the findings of the synthesis. However, the stages at which studies were excluded is detailed in table 1 (page 52) and the narrative on page 10. |
| **10** | Rationale for appraisal | Describe the rationale and approach used to appraise the included studies or selected findings *(e.g. assessment of conduct (validity and robustness), assessment of reporting (transparency), assessment of content and utility of the findings).* | See table 1, pages 10-11, the quality assessment prompts on the data extraction form (appendix) and the narrative concerning each subset of included studies (p. 13-14, pp. 17-19, p.28 & pp.32-3). |
| **11** | Appraisal items | State the tools, frameworks and criteria used to appraise the studies or selected findings *(e.g. Existing tools: CASP, QARI, COREQ, Mays and Pope* [[25](http://www.ncbi.nlm.nih.gov/pmc/articles/PMC3552766/#B25)]*; reviewer developed tools; describe the domains assessed: research team, study design, data analysis and interpretations, reporting).* | Mary-Dixon Woods et al. “prompts” (data extraction form, appendix). Reference: Dixon-Woods M, Cavers D, Agarwal S, Annandale E, Arthur A, et al. (2006) Conducting a critical interpretive synthesis of the literature on access to healthcare by vulnerable groups. BMC Med Res Methodol 6: 35. |
| **12** | Appraisal process | Indicate whether the appraisal was conducted independently by more than one reviewer and if consensus was required. | Appraisal conducted independently by lone workers. Consensus not sought. Studies were more likely to be excluded where findings did not “fit” rather than on quality grounds alone. Previous studies have shown very poor inter-rater reliability on quality appraisal of qualitative studies. Reference: Campbell R, Pound P, Morgan M, Daker-White G, Britten N, et al. (2011) Evaluating meta-ethnography: systematic analysis and synthesis of qualitative research. Health Technol Assess 15: 1-164. |
| **13** | Appraisal results | Present results of the quality assessment and indicate which articles, if any, were weighted/excluded based on the assessment and give the rationale. | No articles were excluded on the basis of quality assessment alone. However, the narrative clearly states which articles were considered to be seriously flawed: Ref. 39 (page 14); Refs. 47 & 52 (page 18). |
| **14** | Data extraction | Indicate which sections of the primary studies were analysed and how were the data extracted from the primary studies? *(e.g. all text under the headings “results /conclusions” were extracted electronically and entered into a computer software).* | The whole manuscript was read and verbatim findings were extracted onto electronic versions of the extraction/appraisal form (pp.10-11). |
| **15** | Software | State the computer software used, if any. | Excel, Word and EndNote (p.10) |
| **16** | Number of reviewers | Identify who was involved in coding and analysis. | All authors except CS (p.11-12 and statement on contributions) |
| **17** | Coding | Describe the process for coding of data *(e.g. line by line coding to search for concepts).* | See page 11 – the whole articles were read for “findings” – as distinct from “data” |
| **18** | Study comparison | Describe how were comparisons made within and across studies *(e.g. subsequent studies were coded into pre-existing concepts, and new concepts were created when deemed necessary).* | “iteratively re-organising tables of concepts or factors” (p.12) |
| **19** | Derivation of themes | Explain whether the process of deriving the themes or constructs was inductive or deductive. | Inductive, following organisation of tables and searches for “third order interpretations” (p.12). But note that this was not a “thematic” synthesis. |
| **20** | Quotations | Provide quotations from the primary studies to illustrate themes/constructs, and identify whether the quotations were participant quotations of the author’s interpretation. | At data extraction, distinction was made between “findings” (which in a minority of cases were participant quotations – but see above) and our interpretations of the same. In tables 2-6, the exact text used in the original articles has been preserved. Tables 7-11 have more in the way of our own interpretations, although original quotes are retained where there was no development beyond the original concepts. |
| **21** | Synthesis output | Present rich, compelling and useful results that go beyond a summary of the primary studies (e.g. *new interpretation, models of evidence, conceptual models, analytical framework, development of a new theory or construct).* | Tables 7-11, figures 2 & 3, and subsection “The moral and emotional foundations of patient safety in primary care,” pp. 39-42. |
