# Supplementary material for: Blame the Patient, Blame the Doctor or Blame the System? A Meta-Synthesis of Qualitative Studies of Patient Safety in Primary Care
Source: PLoS One. 2015 Aug 5;10(8):e0128329. doi: 10.1371/journal.pone.0128329 (PMC4526558; doi:10.1371/journal.pone.0128329)
Supplement: S1 Appendix — (DOCX) [file pone.0128329.s002.docx]

**Appendix 1 - Data Extraction form: Meta-synthesis of qualitative studies of patient safety in primary care**

| **Paper ID** |  | | |
| --- | --- | --- | --- |
| Date |  | | |
| Extracted by |  | | |
| Title of Article |  | | |
| Publication Date |  | | |
| Type of article |  | | |
| **Study details** | Location / Country |  | |
|  | Research question / Objectives |  | |
| **Discipline** | Or multi-disciplinary |  | |
| **Quality** | Are the aims and objectives clearly stated? | | Yes / No |
|  | Is the design clearly specified and appropriate? | | Yes / No |
|  | Do the researchers provide a clear account of the process through which findings were produced? | | Yes / No |
|  | Do the researchers display enough data to support their interpretations and conclusions? | | Yes / No |
|  | Is the method of analysis appropriate and adequately explicated? | | Yes / No |
|  | QUALITY? | Excellent / Acceptable / Unacceptable | |
|  | If ‘Unacceptable,’ Why? |  | |
| **Participants** | Population |  | |
|  | Age |  | |
|  | Age (mean/range) |  | |
|  | Gender (M/ F) |  | |
|  | Ethnicity |  | |
|  | Recruitment / sampling (inclusion criteria, response rates, diffs. between responders and non-responders) |  | |
| **Data Collection** | Methods |  | |
|  | Trustworthiness |  | |
| **Data Analysis** | Method |  | |
| Is it primarily descriptive? | |  | |
| Is it an ‘exploratory’ study, pilot or protocol? | |  | |
| How are results presented? | |  | |
| **Main findings**: Theme / Concept #1  Outline in detail, using author’s own words (in quotation marks with page references) in describing main findings.  If you make your own interpretations of the data/findings, record these but clearly label as your interpretation | |  | |
| **Main findings**: Theme / Concept #2 | |  | |
| **Main findings**: Theme / Concept #3 | |  | |
| **Main findings**: Theme / Concept #4 | |  | |
| **Main findings**: Theme #5 | |  | |
| **Main findings**: Theme #6 | |  | |
| **Main findings**: Theme #7 | |  | |
| **Main findings**: Theme #8 | |  | |
| **Memos (i.e. implications for developing concepts and theories)** | |  | |
| **Meta-narratives (i.e. assumed concepts and theories)** | |  | |
| **Comments (Limitations, reviewer comments, etc.)** | |  | |
| **References** – Possible new | |  | |
| **References** – For Background | |  | |
